# EEG features in late-onset epilepsy: possible correlation with cognitive impairment

**DOI:** 10.1093/braincomms/fcag067

**Published:** 2026-03-14

**Authors:** Lu Lu, Peiyu Wang, Xintong Wu, Weixi Xiong, Josemir W Sander, Jiani Chen, Dong Zhou

**Affiliations:** Department of Neurology, West China Hospital, Sichuan University, Chengdu 610041, China; Department of Neurology, West China Hospital, Sichuan University, Chengdu 610041, China; Department of Neurology, West China Hospital, Sichuan University, Chengdu 610041, China; Department of Neurology, West China Hospital, Sichuan University, Chengdu 610041, China; Department of Neurology, West China Hospital, Sichuan University, Chengdu 610041, China; Queen Square Institute of Neurology, London WC1N 3BG, UK; Chalfont Centre for Epilepsy, Chalfont St Peter SL9 0RJ, UK; Department of Neurology, West China Hospital, Sichuan University, Chengdu 610041, China; Department of Neurology, West China Hospital, Sichuan University, Chengdu 610041, China

**Keywords:** seizure, elderly, Alzheimer's disease, dementia

## Abstract

Late-onset epilepsy (LOE) increases the risk of cognitive impairment and is associated with dementia in a bidirectional manner. Scalp EEG is a potential tool for assessing this relationship, but this has not been fully explored. This cross-sectional study reviewed people who had undergone 24-h EEG monitoring at West China Hospital. Individuals with epilepsy whose seizure onset ≥55 years and older healthy controls were included. Diagnosis of cognitive impairment was established at the time of EEG using standardized neuropsychological metrics. EEGs were reviewed and annotated independently by two neurophysiologists. EEG features were extracted according to the standardized computer-based organized reporting of EEG. Potential clinical, MRI, and EEG risk factors of cognitive impairment were analysed in ordinal and binomial regression models. Least absolute shrinkage and selection operator model was used to select the variables most discriminative of cognitive impairment. Among the screened individuals (*n* = 8318), eligible participants with LOE (*n* = 287) and healthy controls (*n* = 132) were included; median age 65 (55–85) years; female 38%. Epilepsy aetiology was unknown in 177 (62%) participants. Structural aetiology was the most common in epilepsies (93%) with a definite aetiology (*n* = 110). Interictal epileptiform discharges were detected in 56% of participants with LOE and were primarily left temporal. Bitemporal interictal epileptiform discharges were found predominantly (38%) in those of an unknown aetiology. Focal slowing was found in 56%, and generalized slowing in 13% of participants with LOE. EEG markers of temporal neural hyperexcitability, including temporal intermittent rhythmic delta activity (TIRDA) and anterior temporal epileptiform discharges, were associated with cognitive impairment. In LOE of unknown aetiology, bilateral anterior temporal epileptiform discharges were the most discriminative indicator of cognitive impairment [odds ratio 53.280, 95% confidence interval (CI) 11.359–249.917]. The least absolute shrinkage and selection operator model achieved an area under the receiver operating characteristic curve of 0.869 (95% CI 0.813–0.925). In LOE with a definite aetiology, TIRDA was associated with cognitive impairment. In this study, LOE was associated with specific EEG patterns. Signatures of temporal hyperexcitability on EEG might be related to cognitive impairment in LOE, especially when presented in both hemispheres. These results also suggested that LOE of an unknown aetiology might have a neurodegenerative origin, similar to Alzheimer's disease. Future longitudinal studies should explore the role of temporal hyperexcitability and other EEG features in the bidirectional link between LOE and dementia.

## Teaser

Lu *et al*. thoroughly described EEG features in people with new-onset epilepsy in elderly ages, using EEG from 24-h monitoring. They found special slowing and epileptiform patterns in temporal regions, which were associated with cognitive impairment. The results suggested that temporal hyperexcitability might link epilepsy and dementia in the elderly.

## Graphical Abstract

**Graphical Abstract:**
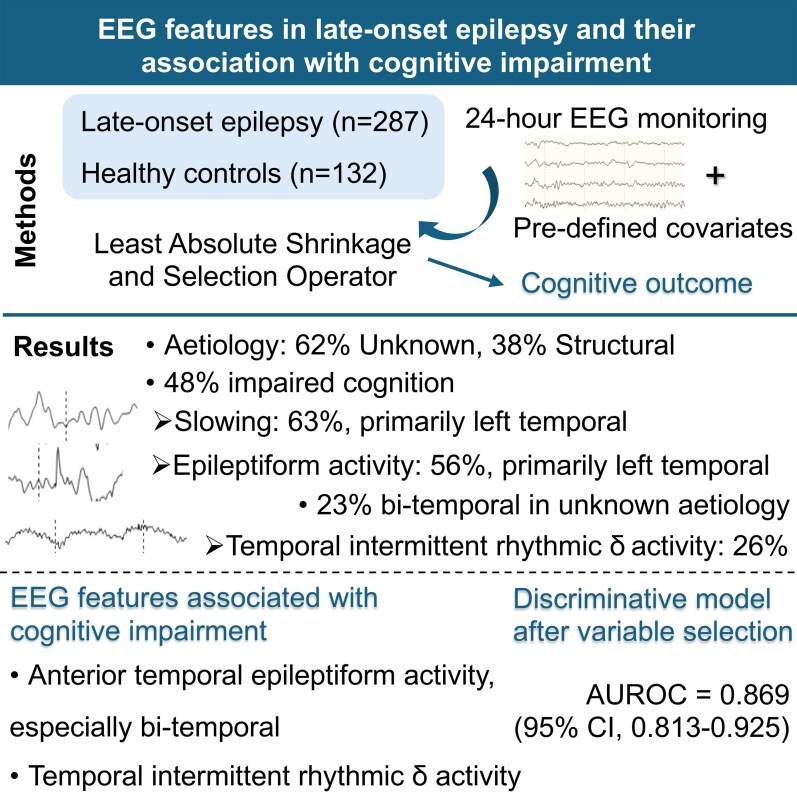

## Introduction

Epilepsy is the third most common neurological disorder in older people, with an increasing incidence after 50 years of age.^1,2^ New-onset epilepsy in older adults [late-onset epilepsy (LOE)] has been widely discussed as a distinct epilepsy subtype. A substantial proportion of LOEs is secondary to late-life brain insults (e.g. stroke, traumatic brain injury, encephalitis, etc.), and up to one third of LOEs have an unknown aetiology (LOEU), which is hypothesized to have an undetected neurodegenerative or cerebrovascular origin.^2-5^

LOE is known for the concomitant risk of mild cognitive impairment and dementia. Data from a population-based study reported an almost threefold higher risk of dementia in people with LOE.^6^ Accumulating evidence has supported a bidirectional relationship between dementia and LOE, especially in LOEU.^7-9^ People with Alzheimer's disease have a greater chance of developing clinical seizures and epileptiform activities on EEG, which are associated with accelerated cognitive decline.^10^ The underlying mechanism remains debated. Some suggested that neural hyperexcitability could account for this association.^11,12^ Recent reports have emphasized treating epileptiform activities and seizures in Alzheimer's disease.^13^ Vice versa, detection and intervention in LOEs at a higher risk of cognitive impairment in the early stage would also be critical, yet no reliable biomarkers are available.

EEG is a widely acknowledged, non-invasive tool for assessing cerebral function. It has been proven useful in evaluating cognitive outcomes of both epilepsy and dementia. Generalized and focal background slowing on EEG are validated markers in Alzheimer's disease and other dementing illnesses.^14,15^ Interictal epileptiform discharges (IEDs) are associated with cognitive comorbidities in epilepsy.^16,17^ EEG is also valuable in detecting subclinical epileptiform activity, in forms of IEDs or temporal intermittent rhythmic delta activity (TIRDA), and predicting seizure development in Alzheimer's disease.^10-12,18^ However, the role of EEG in LOE remains largely unexplored. Most studies on LOE reported electrophysiological findings from a limited cohort using routine EEG, potentially missing epileptiform activity in this population.^19-23^

We used data from long-term (24-h) EEG monitoring to characterize the EEG signatures in LOE. We suggest that specific EEG patterns may be associated with cognitive impairment in LOE, and that these non-invasive markers could help stratify individuals at higher risk.

## Materials and methods

### Study design

This was a cross-sectional cohort study at West China Hospital, Sichuan University. This study was conducted in line with the principles of the World Medical Association Declaration of Helsinki. The Ethical Committees of West China Hospital approved this study (2024[1834]). All individuals provided written informed consent at admission. We followed the Strengthening the Reporting of Observational Studies in Epidemiology (STROBE) Reporting Guidelines.^24^

### Study cohort

We retrospectively assessed individuals with LOE who underwent 24-h video-EEG (VEEG) monitoring at West China Hospital between December 2021 and May 2025. Epilepsy was diagnosed according to the International League Against Epilepsy (ILAE) definition.^25^ LOE was considered when participants had their first unprovoked epileptic seizure after the age of 55. All LOEs were included, regardless of the aetiology. To characterize the EEG features of LOE, and to avoid potential effects from acute brain insults and other neuropsychological disorders other than epilepsy, participants meeting the following criteria were excluded: (i) acute phase of major systemic diseases (e.g. severe pneumonia, myocardial infarction, septic shock) within 6 months before VEEG recording, (ii) acute phase of neurological disorders (e.g. stroke, brain tumour, traumatic brain injury with loss of consciousness, meningitis/encephalitis, multiple sclerosis), or brain surgery within 6 months before VEEG recording; (iii) history of major mental disorders (e.g. major depressive disorder, psychotic disorder, substance misuse), following diagnostic and statistical manual of mental disorders, 5th edition (DSM-5).^26,27^

Age- and gender-matched healthy controls(HC) were recruited during the same study period. These were individuals who underwent diagnostic recording and turned out to have non-epileptic events, mainly syncope or vertigo.

Participants with LOE were divided into two groups according to aetiology: LOE of unknown aetiology (LOEU) and LOE with a definite aetiology (LOED). LOEU was defined as LOE without possible secondary causes of seizures. Under this definition, participants would be excluded as LOEU if they had a previous diagnosis of stroke, cerebral infection, brain tumour, brain surgery, or neurotoxin exposure. The classification of LOEU was ascertained through comprehensive investigations with MRI, VEEG, and CSF testing. Participants who didn't meet the definition of LOEU were categorized as LOED. Two experienced neurologists established the aetiology diagnosis (L.L. and D.Z.).

### Outcome

According to individual cognitive performance, participants with LOE were further divided into two groups: those with impaired cognition (LOE-CI) and those with normal cognition (LOE-CN). Cognitive impairment was defined as (i) having a diagnosis of cognitive impairment based on the DSM-5 approach at the time of admission or (ii) having Mini-Mental State Examination (MMSE) scores below 26 or Montreal Cognitive Assessment (MoCA) scores below the age- and education-adjusted norm recommended in Chinese guidelines at the time of VEEG.^28,29^ MMSE, MoCA, and other neuropsychological tests required to establish the diagnosis of cognitive impairment were performed at admission by trained neuropsychologists from West China Hospital.

### VEEG recording

VEEG monitoring was performed on each individual using the Nihon Kohden digital video-EEG 1200 instrument (Nihon Kohden, Japan). Scalp electrodes were placed according to the International 10–20 system, with the addition of inferior temporal lobe electrodes FT9/FT10 and TP9/TP10.

### EEG variables extraction

VEEGs were independently reviewed by two neurophysiologists. EEG variables were extracted according to the ‘background activity’ and ‘interictal findings’ categories under the standardized computer-based organized reporting of EEG (SCORE) standard.^30^ Focal features were further divided into temporal and extratemporal based on their localization, as LOEs are predominantly temporal lobe epilepsy.^21,22,31^

The localization of IEDs was determined based on the electrode sites where the peak negativity is observed (i.e. the location of maximum negativity). IRDA was marked with a specific interest in the temporal regions (TIRDA). The initial annotations will be jointly reviewed by two neurophysiologists (J.C. and W.X.) to reach a consensus conclusion. Disagreement would be mediated by a third reviewer (P.W.) who did not participate in the first-round evaluation.

### Covariates

Demographic data, clinical characteristics, and neuroimaging results were extracted by two physicians using a standardized case report form. The classification of epilepsies and seizures was reviewed and determined by two specialists (P.W. and L.L.) following ILAE guidelines.^32^ To investigate the potential effect of cerebral small-vessel disease (CSVD) on cognition, we classified several MRI markers as CSVD signs, including small subcortical infarcts, white matter hyperintensities of presumed vascular origin, perivascular spaces, and cerebral microbleeds. One experienced neuroradiologist (L.L.) reviewed the MRI for evidence of CSVD. Hamilton Depression Rating Scale (24-item) and Hamilton Anxiety Rating Scale were used to measure anxiety and depression.

The choice of covariates was based on a prior structured search of evidence and clinical experience. A systematic literature search was conducted in PubMed, Ovid-Embase, Web of Science, Scopus, and a Chinese online database (CNKI), using the following entry terms: ‘seizure’, ‘epilepsy’, ‘cognitive impairment’, ‘cognition’, ‘dementia’, ‘risk’, and ‘association’. Accordingly, variables included as covariates were individual demographic details (age, sex, years of education), previous medical history (history of stroke, traumatic brain injury, brain surgery, brain tumour, encephalitis and cerebral infection), cardiovascular risk factors (hypertension, diabetes mellitus, hyperlipidaemia, smoker, and obesity), epilepsy characteristics (epilepsy duration, history of bilateral tonic-clonic seizure, having active seizures during the 6 months before VEEG), medications [number of antiseizure medications (ASMs)], MRI findings (cerebral atrophy and CSVD signs).^9,31-37^ In addition to these predefined variables, any other characteristics that were found to be associated with cognitive impairment in the univariate analyses (regular alcohol intake, mild depression and/or anxiety) would also be considered.^9,33-39^

### Statistical analysis

We used *χ*^2^ tests, Fisher exact tests, Mann–Whitney U-tests, Kruskal–Wallis tests, ANOVA, and independent *t*-tests to compare variables among different groups when appropriate. Multiple imputation by chained equations was performed to estimate missing data and generate 10 complete datasets for regression analysis and model development.^40^ Ordinal logistic regression was performed to examine clinical and EEG variables associated with the outcomes (e.g. controls, LOE-CI, and LOE-CN). Binomial logistic regression was performed to explore potential risk factors associated with cognitive impairment. Least absolute shrinkage and selection operator (LASSO) regression was performed in each of the imputed datasets for variable selection, and the variables that preserved a non-zero coefficient in the LASSO model in no less than eight imputed datasets were accepted for inclusion in the multivariate binomial logistic regression to construct the final model. The area under the receiver operating characteristic curve (AUROC), sensitivity, specificity, positive predictive value, negative predictive value, and accuracy rate were calculated to examine the performance of the final model. Estimates of variable effect in regression analyses and metrics of model performance in each of the imputed datasets were pooled using Rubin's rules.^41^ Chi-squared tests, Fisher exact tests, Mann–Whitney U-tests, Kruskal–Wallis tests, ANOVA, and independent *t*-tests were performed using SPSS statistics software (version 26.0). Ordinal logistic regression, binomial logistic regression, and LASSO regression were performed using R software (version 4.4.1). Visualization of focal slowing and IED locations was performed in MATLAB (MathWorks) using a function for creating brain plots.^42^ Statistical tests were two-tailed, and *α* = 0.05 was used to determine significance. Multiplicity generated by the ordinal and binomial regression analyses was controlled using the adaptive linear step-up approach to control the false discovery rate (FDR).^43^ Odds ratios (ORs) are given with a 95% confidence interval (CI). Categorical variables are shown in proportions, and continuous variables are shown in mean [standard deviation (SD)] or median [interquartile range (IQR)].

## Results

**Figure 1:**
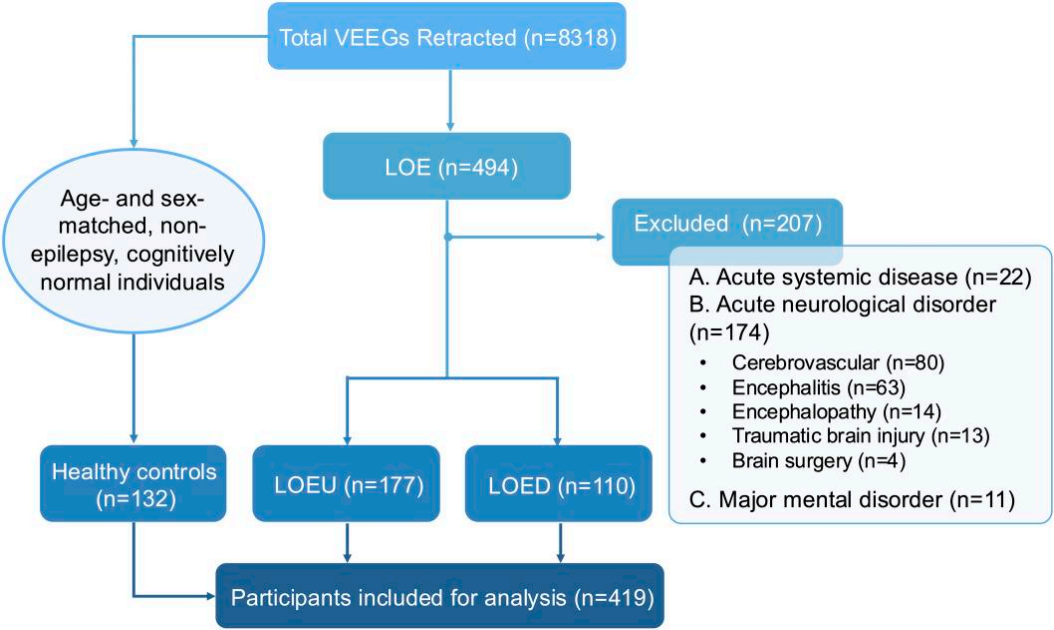
**Inclusion flow chart. In sum, 494 people with LOE were identified from the retracted VEEGs**. Two hundred and seven people with LOE were excluded according to the pre-specified criteria. Non-epilepsy, cognitively normal individuals without psychological or neurological disorders were identified from the retracted VEEGs and included as healthy controls. A total of 419 participants were included for further analysis. VEEG, video electroencephalography; LOE, late-onset epilepsy; LOED, late-onset epilepsy with a definite aetiology; LOEU, late-onset epilepsy of unknown aetiology.

Eight thousand three hundred eighteen individuals were screened, of whom 494 had LOE. Two hundred and eighty-seven participants who met the inclusion criteria were included, as shown in Fig. 1. Median age was 65 (55–85) years, and 178 (62%) were male. The indications for VEEG monitoring included diagnosis (*n* = 116), evaluation of treatment response (*n* = 89), and characterization of known epilepsy (*n* = 82). One hundred and thirty-two people were enrolled as controls. Their discharge diagnoses included syncope (*n* = 98), vertigo (*n* = 14), autonomic dysfunction (*n* = 12), myoclonus (*n* = 5), dystonia (*n* = 1), hemifacial spasm (*n* = 1), and obstructive sleep apnoea (*n* = 1). Baseline demographic and clinical characteristics are shown in Table 1. Age, sex, years of education, and frequency of common comorbidities were comparable between groups. Neuropsychological comorbidities were more common in participants with LOE compared with those in controls.

**Table 1:** Baseline characteristics

	LOE (*n* = 287)	HC (*n* = 132)	Overall (*n* = 419)	*P* ^a^
Demographics				
Age, median (IQR)	65 (11)	64 (10)	65 (10)	0.068
Female, *n* (%)	109 (38)	62 (47)	171 (41)	0.088
Occupation				
Physical labour, *n* (%)	67 (23)	24 (18)	91 (22)	
Mental labour, *n* (%)	31 (11)	14 (11)	45 (11)	
Retired/unemployed, *n* (%)	189 (66)	94 (71)	283 (67)	
Education^b^				
Uneducated, *n* (%)	16 (6)	7 (5)	23 (5)	
Primary school, *n* (%)	53 (18)	31 (23)	84 (20)	
Junior high, *n* (%)	97 (34)	27 (20)	124 (30)	
Senior high, *n* (%)	60 (21)	31 (23)	91 (22)	
College, *n* (%)	60 (21)	35 (27)	95 (23)	
Years of education, median (IQR)	9 (3)	12 (10)	9 (6)	
Personal history				
Smoking, *n* (%)	64 (22)	32 (24)	96 (23)	
Regular alcohol consumption, *n* (%)	50 (17)	26 (20)	76 (18)	
BMI, mean (SD)^c^	23.39 (3.11)	23.43 (3.04)	23.40 (3.08)	
Overweight, *n* (%)	115 (40)	59 (45)	174 (42)	
Comorbidity				
High blood pressure, *n* (%)	109 (38)	41 (31)	150 (36)	
Diabetes mellitus, *n* (%)	46 (16)	20 (15)	66 (16)	
Hyperlipidaemia, *n* (%)	40 (14)	16 (12)	56 (13)	
Coronary heart disease, *n* (%)	19 (7)	8 (6)	27 (6)	
Mild depression and/or anxiety, *n* (%)	37 (13)	4 (3)	41 (10)	**0**.002*
Sleep disorder, *n* (%)	71 (25)	14 (11)	85 (20)	< 0.001*
All-cause dementia, *n* (%)	13 (4)	0	0	**0**.012*
Medical history				
History of stroke, *n* (%)	33 (11)	0	33 (8)	< 0.001*
History of head injury, *n* (%)	16 (5)	0	16 (4)	**0**.004*
History of cerebral infection/encephalitis, *n* (%)	8 (3)	0	8 (2)	0.061
History of cerebral surgery, *n* (%)	28 (10)	0	28 (7)	< 0.001*
Brain tumour, *n* (%)	17 (6)	0	17 (4)	**0**.006*
Epilepsy aetiology				
Structural, *n* (%)	102 (36)			
Metabolic, *n* (%)	6 (2)			
Immune, *n* (%)	2 (1)			
Unknown, *n* (%)	177 (61)			

BMI, body mass index. ^a^Only *P* < 0.1 are shown. *P*-values showing statistical significance (<0.05) were bolded and followed by asterisks (*). ^b^Incomplete data for education. Sample sizes with complete education: LOE (*n* = 286), HC (*n* = 131). ^c^Incomplete data for BMI. Sample sizes with complete BMI: LOE (*n* = 281), HC (*n* = 132).

### Demographic and clinical features

Among the participants with LOE, 177 (62%) were classified as LOEU, while 110 (38%) had a definite aetiology (LOED). The causes of epilepsy included stroke (*n* = 35), trauma (*n* = 18), brain tumour (*n* = 15), encephalitis (*n* = 14), lesional MRI without related medical history (*n* = 20), and possible metabolic (*n* = 6) and immune aetiology (*n* = 2).

The epilepsy- and EEG-related features among the LOEU, LOED, and HC groups are shown in Table 2. Median epilepsy duration was 12 months, and the median age of epilepsy onset was 63 years. Epilepsy duration, age of epilepsy onset, and seizure frequency were comparable between the LOEU and LOED groups. A significantly higher proportion of participants in the LOEU group were treatment-naive than in the LOED group (*P* < 0.001, *χ*^2^ test). Participants with LOED were more frequently receiving polytherapy for seizure control (*P* < 0.001, *χ*^2^ test). The most prescribed ASMs were levetiracetam, sodium valproate, and oxcarbazepine. MRI revealed that LOED participants were more likely to show CSVD signs and encephalomalacia focus than their LOEU counterparts.

**Table 2:** Epilepsy, medication, MRI, and EEG characteristics

	LOEU (*n* = 177)	LOED (*n* = 110)	HC (*n* = 132)	*P* ^a^
Age at onset, median years (IQR)	64 (11)	61 (10)		
Cognitive impairment, *n* (%)	76 (43)	63 (57)		**0**.021*
MMSE, median (IQR)	25 (8)	24 (3)	29 (1)	**<0**.001*
MoCA, median (IQR)	19 (10)	15 (6)	26 (1)	**<0**.001*
Seizure type				
Focal, preserved consciousness, *n* (%)	40 (23)	33 (30)		
Focal, impaired consciousness, *n* (%)	84 (47)	58 (53)		
Focal to bilateral tonic-clonic, *n* (%)	72 (41)	49 (45)		
Generalized, *n* (%)	2 (1)	1 (1)		
Unknown, *n* (%)	19 (11)	6 (5)		
Seizure frequency^b^				
Seizure free for more than 2 years, *n* (%)	14 (8)	18 (16)		
Seizure free for more than 1 year, *n* (%)	7 (4)	3 (3)		
Per year, *n* (%)	28 (16)	17 (15)		
Per several months, *n* (%)	20 (11)	18 (16)		
Per month, *n* (%)	54 (31)	28 (26)		
Per week, *n* (%)	21 (12)	10 (9)		
Per day, *n* (%)	31 (18)	16 (15)		0.068
ASM treatment				
Treatment naïve, *n* (%)	91 (51)	32 (29)		**<0**.001*
Current number of ASMs, median (IQR)	0 (1)	1 (2)		**0**.001*
Monotherapy, *n* (%)	56 (32)	40 (36)		
Polytherapy, *n* (%)	20 (11)	34 (31)		**<0**.001*
Epilepsy localization				
Left temporal, *n* (%)	56 (32)	37 (34)		
Right temporal, *n* (%)	38 (21)	22 (20)		
Bitemporal, *n* (%)	25 (14)	5 (5)		
Frontal, *n* (%)	4 (2)	28 (25)		
Parietal, *n* (%)	2 (1)	7 (6)		
Occipital, *n* (%)	0	1 (1)		
Unknown, *n* (%)	52 (29)	10 (9)		
MRI				
Performed, *n* (%)	151 (85)	102 (93)	98 (74)	
Pathological, *n* (%)	89 (59)	99 (97)	48 (49)	**<0**.001*
Hippocampal sclerosis, *n* (%)	2 (1)	11 (11)	0	**<0**.001*
Generalized cerebral atrophy, *n* (%)	54 (36)	42 (41)	22 (22)	**0**.015*
CSVD signs, *n* (%)	33 (22)	50 (49)	28 (29)	**<0**.001*
Vascular malformation, *n* (%)	5 (3)	10 (10)	3 (3)	0.066
Encephalomalacia, *n* (%)	0	59 (54)	1 (1)	**<0**.001*
EEG				
Slowing, *n* (%)	98 (55)	82 (75)	35 (27)	**<0**.001*
IED, *n* (%)	99 (56)	63 (57)	15 (11)	**<0**.001*

^a^Only *P* < 0.1 are shown. *P*-values showing statistical significance (<0.05) were bolded and followed by asterisks (*). ^b^Incomplete data for seizure frequency. Sample sizes with complete education: LOEU (*n* = 175), LOED (*n* = 110).

### EEG features in LOE

Abnormal EEGs were identified in 223 participants with LOE (78%) and 43 controls (33%). No significant differences were noted between LOED and LOEU groups in background characteristics, including the frequency, amplitude, symmetry, regulation, and modulation of the posterior dominant rhythm. Detailed EEG characteristics are presented in Supplementary Table 1.

**Figure 2:**
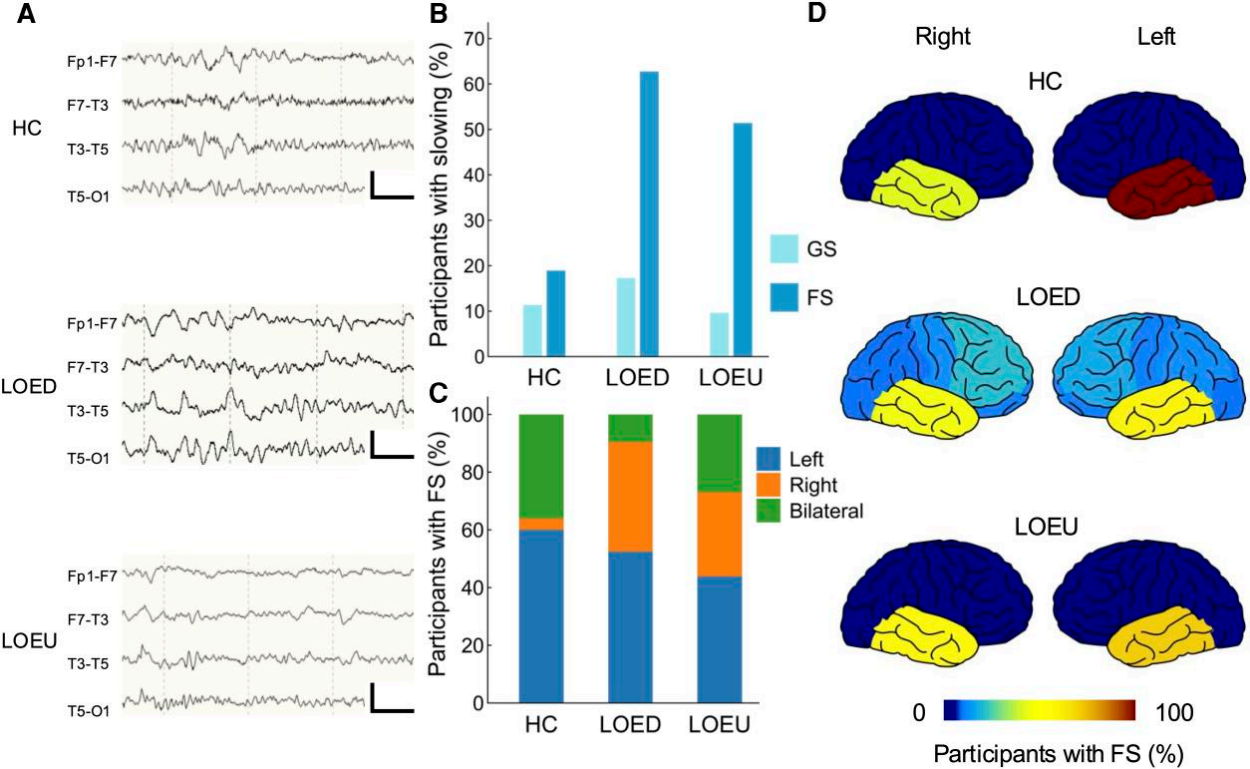
**Characteristics of slowing on EEG in LOE**. (**A**) Representative examples of left temporal slowing in HC (*n* = 132), LOED (*n* = 110), and LOEU (*n* = 177) participants. EEG channels: Fp1-F7, F7-T3, T3-T5, T5-O1 (top to bottom). Calibration bars: 50 µV, 500 ms. (**B**) Proportions of participants showing slowing on EEG. (**C**) Distribution of focal slowing by lateralization, standardized to individual participant. (**D**) Schematic demonstration of focal slowing location. Colour bar represents the proportion of participants with focal slowing at the indicated location. EEG, electroencephalography; FS, focal slowing; GS, generalized slowing; HC, healthy control; LOED, late-onset epilepsy with a definite aetiology; LOEU, late-onset epilepsy of unknown aetiology.

Around half of the participants exhibited slowing on EEG (Fig. 2). Generalized slowing was present in 36 participants with LOE (20%) and 15 controls (11%). Focal slowing was found in 63% LOED participants and 51% LOEU participants (*P* = 0.067, *χ*^2^ test). In controls, focal slowing was observed in 27% and was exclusively temporal. In LOEUs, focal slowing was primarily temporal (98%). In LOEDs, focal slowing was also primarily temporal (91%), but also occurred in the frontal (33%), central (20%), parietal (19%), and occipital (16%) regions. Slowing was concordant with the presumed epileptic focus in 62 (63%) LOEU and 50 (61%) LOED participants showing slowing on EEG.

**Figure 3:**
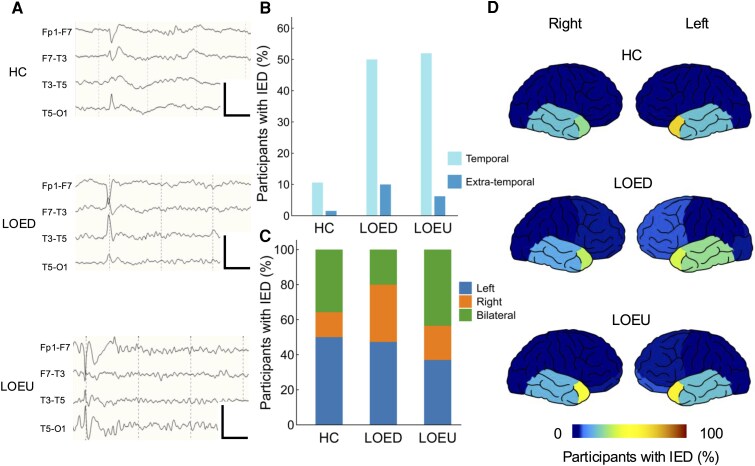
**Characteristics of epileptiform activity on EEG in LOE**. (**A**) Representative examples of left temporal IED in HC (*n* = 132), LOED (*n* = 110), and LOEU (*n* = 177) participants. EEG channels: Fp1-F7, F7-T3, T3-T5, T5-O1 (top to bottom). Calibration bars: 100 µV, 500 ms. (**B**) Proportions of participants showing temporal IED or extratemporal IED on EEG. (**C**) Distribution of IED by lateralization, standardized to individual participant. (**D**) Schematic demonstration of IED location. Colour bar represents the proportion of participants with IED at the indicated location. EEG, electroencephalography; HC, healthy control; IED, interictal epileptiform discharge; LOED, late-onset epilepsy with a definite aetiology; LOEU, late-onset epilepsy of unknown aetiology.

IEDs were seen in 162 participants with LOE (56%) and 15 (11%) in controls (*P* < 0.001, *χ*^2^ test) (Fig. 3). The most common morphology of IEDs was sharp wave (78%), followed by spike (15%) and sharp-and-slow-wave (6%). IEDs were also predominantly located in the temporal region (90%). A substantial proportion of IEDs were seen in both temporal regions. It was higher in LOEU (38%) than in LOED (17%) of participants with IEDs (*P* = 0.015). FT9/FT10 electrodes were the most frequently reported location for maximum IEDs in LOE (54%), followed by T3/T4 (26%) and F7/F8 (21%). IEDs were concordant with the epileptic focus in 78 (79%) LOEU and 56 (89%) LOED participants showing IEDs on EEG (*P* = 0.135, *χ*^2^ test).

**Figure 4:**
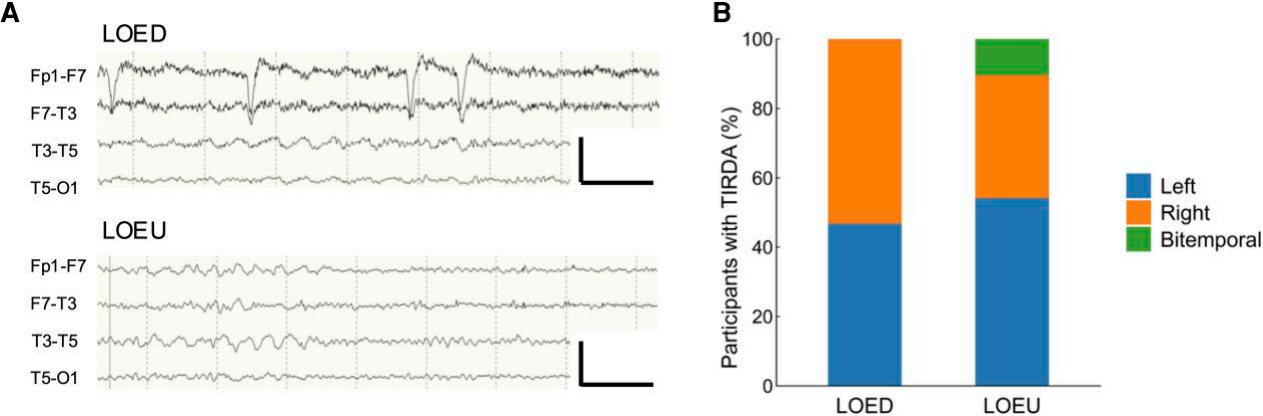
**Characteristics of TIRDA in LOE**. (**A**) Representative examples of left TIRDA in HC (*n* = 132), LOED (*n* = 110), and LOEU (*n* = 177) participants. EEG channels: Fp1-F7, F7-T3, T3-T5, T5-O1 (top to bottom). Calibration bars: 100 µV, 1 s. (**B**) Distribution of TIRDA by lateralization, standardized to individual participant. LOED, late-onset epilepsy with a definite aetiology; LOEU, late-onset epilepsy of unknown aetiology; TIRDA, temporal intermittent rhythmic delta activity.

Intermittent delta activity was detected in 80 LOEs (28%) with a temporal predominance (98%). TIRDA occurred in 48 (28%) LOEU and 30 (28%) LOED participants (Fig. 4). In LOEU, TIRDA was predominantly left (54%), with 10% bitemporal TIRDA. In LOED, TIRDA was either left (47%) or right (53%) temporal. TIRDA was concordant with the presumed epileptic focus in 77 (99%) LOE participants; one LOED participant had right TIRDA and midline IEDs during EEG recording, whose epileptic focus was presumed to be left parietal. IEDs occurred in 83% LOE participants showing TIRDA. In these participants, TIRDA and epileptiform activity were concordant in 95%.

### Cognitive impairment in LOE

Participants with LOE demonstrated significantly poorer cognitive performance on the MMSE (*P* < 0.001, Mann–Whitney U-test) and MoCA (*P* < 0.001, Mann–Whitney U-test) compared with controls. Forty-eight per cent of the participants with LOE were considered cognitively impaired. This proportion was significantly higher among participants with LOED than those with LOEU (*P* = 0.021, *χ*^2^ test), although they had comparable scores on MMSE and MoCA (Table 2).

### EEG features were associated with cognitive impairment in LOE

Ordinal logistic regression demonstrated that across the controls, LOE-CI, and LOE-CN groups, significant EEG variables included generalized delta slowing, focal delta slowing, and temporal IEDs (Supplementary Table 3). These variables remained significantly associated with cognitive impairment in LOE in the binomial model, after controlling for age, sex, and years of education (Supplementary Table 4).

### EEG correlates with cognitive impairment in LOEU

To focus on the impact of EEG features, we chose participants with LOEU to explore potential EEG risk factors of cognitive impairment. Ordinal logistic regression showed similar outcomes (Supplementary Table 3). Binomial regression was performed between LOEU participants with and without cognitive impairment (LOEU-CI versus LOEU-CN) (Supplementary Table 5). Significant EEG variables after controlling for age, sex, and years of education included overall abnormal EEG (OR 7.837, 95% CI 3.073–19.983, *P*-adjusted = 0.001), slowing on EEG (OR 3.216, 95% CI 1.665–6.212, *P*-adjusted = 0.007), left temporal delta slowing (OR 2.654, 95% CI 1.334–5.277, *P*-adjusted = 0.042), TIRDA (OR 2.657, 95% CI 1.314–5.374, *P*-adjusted = 0.043), and anterior temporal IEDs (left/FT9, OR 13.190, 95% CI 3.933–44.240, *P*-adjusted = 0.001; right/FT10, OR 9.237, 95% CI 2.162–39.462, *P*-adjusted = 0.026; left and right/FT9 and FT10, OR 53.280, 95% CI 11.359–249.917, *P*-adjusted < 0.001).

### EEG correlates with cognitive impairment in LOE with a structural aetiology

The same analyses were performed in the LOED group (Supplementary Table 6). The potential EEG correlate of cognitive impairment in this group was TIRDA (OR 4.330, 95% CI 1.587–11.818, *P* = 0.004), after controlling for age, sex, and years of education. TIRDA remained independently significant after controlling for the presence of temporal lesions on MRI (OR 4.350, 95% CI 1.226–15,434, *P* = 0.025). This association was not significant after correction for multiple comparisons.

### EEG correlates with cognitive impairment in LOE after controlling for confounders

Among the predefined clinical and vascular factors, age was associated with the outcome. Regular alcohol intake and mild depression and/or anxiety were found to be associated with cognitive impairment, in LOE and LOEU, and were included as covariates (Supplementary Tables 3 and 4).

Proportions of participants using sleep aids, antidepressants, antipsychotics, and benzodiazepines were comparable between LOE-CN and LOE-CI groups. The number and type of ASMs used were also similar between the two groups. Dosage and administration of ASMs were in line with current clinical practice standards. Three participants used zonisamide or topiramate, but none were considered cognitively impaired (Supplementary Table 2). On MRI, CSVD signs were more frequently observed in LOE participants with cognitive impairment compared with those without (39 versus 26%, *P* = 0.029, *χ*^2^ test) (Table 2).

The association between cognitive impairment and the above-mentioned EEG features was further established by controlling for the predefined covariates. Generalized delta slowing, left or right temporal focal slowing, TIRDA, and anterior temporal IEDs remained significantly associated with the outcome, in the LOEU group and the overall LOE participants (Table 3).

**Table 3:** EEG characteristics associated with cognitive impairment in LOE, controlling for covariates^a^

	LOE (*n* = 287)^b^		LOEU (*n* = 177)^b^		LOED (*n* = 110)^c^	
	OR^b^	95% CI	OR^b^	95% CI	OR^b^	95% CI
Overall abnormal EEG	6.929	3.174–15.125	13.618	4.386–42.286		
Slowing on EEG	3.370	1.888–6.014	4.039	1.907–8.557	3.496	1.049–11.657
Generalized slowing						
Theta						
Delta			5.780	1.324–25.224		
Focal slowing						
Left temporal						
Theta						
Delta			2.622	1.246–5.519		
Right temporal						
Theta						
Delta	2.949	1.585–5.486	3.234	1.421–7.357	3.259	1.066–9.970
Bilateral temporal						
Both theta						
Delta on either hemisphere						
TIRDA	3.445	1.885–6.295	3.322	1.530–7.209	6.332	1.931–20.764
Left	2.480	1.150–5.345			5.297	1.077–26.054
Right	4.618	1.909–11.170	4.368	1.352–14.112	7.442	1.540–35.965
Left and right					-	-
IED on EEG						
Anterior temporal IEDs						
FT9	11.563	4.230–31.609	19.096	5.041–72.330	6.097	1.042–35.691
FT10	8.307	2.700–25.552	16.780	3.075–91.577		
FT9 and FT10	34.132	9.128–127.633	71.997	13.683–378.827		
Other temporal IEDs						
Left						
Right						
Left and right						
Extratemporal IEDs						
Left						
Right						
Left and right						

^a^Covariates included in the analyses consisted of age, sex, years of education, epilepsy duration, having active seizures 6 months prior to VEEG recording, history of bilateral tonic-clonic seizure, number of ASMs used, mild depression and/or anxiety, cardiovascular risk factors (high blood pressure, diabetes mellitus, hyperlipidaemia, obesity, and CSVD signs on MRI), and history of stroke, traumatic brain injury, brain surgery, brain tumour, CNS infection and encephalitis. ^b^Variables are only if they remained significantly associated with the outcome after being adjusted for multiple comparisons using FDR correction (*P*-adjusted < 0.05). ^c^Variables are shown only if they were significantly associated with the outcome (*P*-unadjusted < 0.05).

### LASSO regression

**Figure 5:**
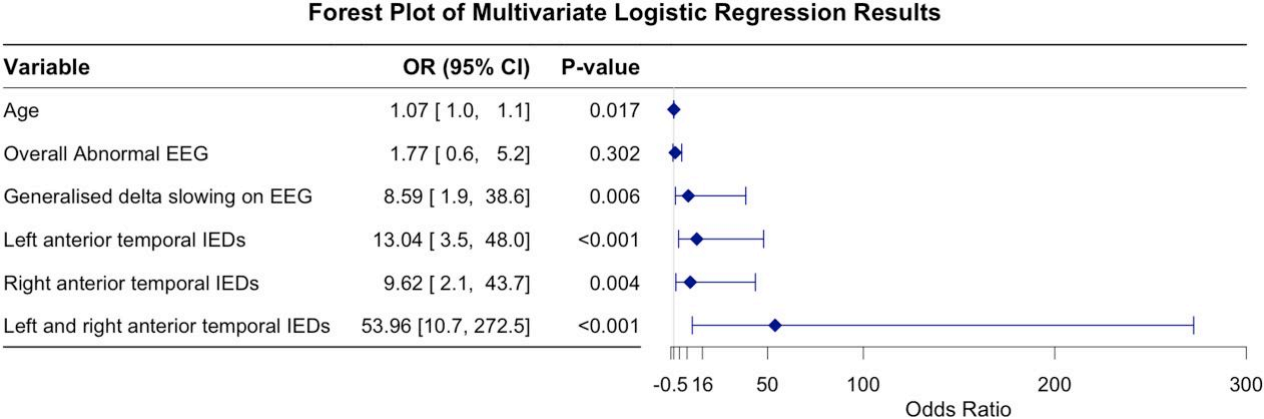
**LASSO logistic regression with LOEU-CI versus LOEU-CN**. Forest plot of the final model of cognitive impairment in LOEU with variables selected by LASSO regression. Generalized delta slowing and anterior temporal IEDs were independently associated with cognitive impairment. Left and right anterior temporal IEDs contributed the most to the model's discriminative performance. Multivariate binomial logistic regression was used for statistical analyses. *N* = 177; experimental unit = individual participant. EEG, electroencephalography; IED, interictal epileptiform discharge; LASSO, least absolute shrinkage and selection operator; LOED, late-onset epilepsy with a definite aetiology; LOEU, late-onset epilepsy of unknown aetiology; LOEU-CN, late-onset epilepsy of unknown aetiology with normal cognition; LOEU-CI, late-onset epilepsy of unknown aetiology with cognitive impairment.

To identify EEG features that best discriminate between participants with and without cognitive impairment in LOEU, we performed LASSO regression to select the optimal combination of variables. All EEG features and covariates were imputed as candidate variables. Age, overall abnormal EEG, generalized delta slowing, and anterior temporal IED were included in the final model, which is well discriminative, with an AUROC of 0.869 (95% CI 0.813–0.925) (Fig. 5; Supplementary Fig. 1).

## Discussion

We report the EEG features in LOE using long-term EEG recordings from a single-centre cohort, including people with LOE and controls. Compared with controls, EEGs of participants with LOE presented distinct slowing and epileptiform discharge profiles with a temporal focus. The LOEU subgroup exhibited an enhanced bilateral anterior temporal localization of EEG abnormalities. Parameters indicating temporal neural hyperexcitability showed proper discriminative performance in the LASSO regression model, with IEDs from bilateral anterior temporal regions being the most significant variable.

IED is an electrophysiological marker of neural hyperexcitability in epilepsy. We detected IEDs in over half of the individuals with LOE. This proportion is higher than that reported in previous studies using routine EEG but similar to that found in long-term EEG recording.^12-19,31,44,45^ IEDs have been reported to be predominantly left temporal in LOEU,^19,21,22,44^ in line with our finding. IEDs were primarily temporal, regardless of aetiology or lesion, with a greater predominance of bilateral anterior temporal IEDs in LOEU.

Clinical observations suggest that cortical and hippocampal hyperactivity are also features of people in the early stages of cognitive disorders (e.g. Alzheimer's disease).^23,46^ These indicators are associated with the severity and progression of cognitive impairment and the development of clinical seizures.^47-50^ Late-onset seizures and epileptiform EEG markers can also appear several years before the onset of dementia symptoms.^10^ Our results revealed that anterior temporal IEDs, especially from bilateral hemispheres, are highly discriminative of LOEU-CI participants. This suggests that people with LOE who showed bilateral network disruptions are more likely to develop cognitive impairment.

The findings also provided evidence of a bidirectional link between LOE and dementia. Accumulation of neurodegenerative pathologies [e.g. phosphorylated tau (pTau) and amyloid-beta (Aβ) in Alzheimer's disease] is deemed the biological basis of this relation.^10^ These pathological deposits generally start from the temporal areas at early stages.^51^ The disrupted neuronal activity would, in turn, facilitate the deposition of pTau and Aβ, and subsequently increase susceptibility to seizures and cognitive impairment.^52^ The bitemporal IEDs are strongly associated with cognitive impairment from our results. This association is particularly notable in LOEs without secondary causes or other significant neurological disorders. As adult-onset epilepsy is generally assumed to be focal, these special EEG patterns in LOEU are reminiscent of the deposition of pathologies in Alzheimer's disease, which is usually symmetrical. Some researchers have proposed that people with Alzheimer's disease and temporal lobe epilepsy may represent a distinct disease subtype.^53^ Another cross-sectional study with 24-h scalp EEG found that bitemporal hyperexcitability was associated with clinical seizures in Alzheimer's disease.^26^ We hypothesize that some LOEU might have a neurodegenerative basis; bilateral anterior temporal IEDs might originate from a shared biological basis with Alzheimer's disease or other dementing disorders.

We found TIRDA in a higher proportion of participants with LOE than in controls, as well as in LOED-CI and LOEU-CI participants compared with their cognitively normal counterparts. TIRDA is another EEG marker of hyperexcitability, indicating mesial temporal regions.^18^ The finding of TIRDA in participants with LOED without a responsible temporal lesion is unexpected. TIRDA is associated with cognitive impairment regardless of lesion location on MRI. This might suggest that people with LOE with temporal hyperexcitability are likely to be cognitively impaired, despite the aetiology of epilepsy.

We found generalized or focal slowing in almost two-thirds of the LOE, a proportion higher than that previously reported.^20^ Temporal theta slowing is considered a normal finding in the ageing brain.^54^ Generalized delta slowing was more frequently observed in LOE-CI than in LOE-CN. Previous reports in dementia have shown that increased diffuse slow activity may indicate diffuse cortical pathologies and extensive network disconnections involved in the development of cognitive impairment.^55^ Impaired network hypersynchrony and altered rhythmic activity could contribute to cognitive decline. Focal slowing has also been reported in Alzheimer's disease and other cognitive complaints. Temporal focal delta slowing was more prevalent in LOE-CI than in LOE-CN, consistent with observations in individuals with dementia.^56,57^ The shift in regional EEG rhythm to slow activity may reflect delayed information transmission among brain regions that underlie cognitive dysfunction. Generalized or temporal slowing might be related to cognitive performance in LOE, but the qualitative nature of the analysis limited the interpretation.

Caution should be taken when generalizing the results in our study to a more general LOE population. Participants with a history of major systemic or neurological disorders within 6 months before VEEG recording were excluded due to concerns about symptomatic seizures. It's critical to select participants with a sustained susceptibility to unprovoked seizures. The existence of a sustained susceptibility to unprovoked seizures is questionable in this group.^39,58-61^ Currently, there is an intense debate over the definition of acute symptomatic seizures.^58,59,62^ As our study was aimed at describing the EEG characteristics and their association with cognitive outcomes in LOE, it would be prudent to avoid the confounding effects of acute brain insults on EEG and cognitive function, which requires a more carefully selected cohort.^63^

### Limitations

Our study has several limitations. Firstly, one primary limitation would be the underrepresentation of structural LOE, particularly those of cerebrovascular origin.^2,64^ This has made our cohort less comparable to other retrospective studies, where a higher proportion of cognitive dysfunction in LOE was reported.^65^ Similarly, we did not identify any significant association between cognitive impairment and vascular risk factors, which was reported in previous studies.^9,32^ While we have conducted separate analyses in LOEs with different aetiology to improve generalizability, the results in the LOED group should be interpreted with caution.

Secondly, participants were enrolled at a single-centre EEG monitoring unit, and a considerable proportion of LOE participants were treatment-naive at baseline. The potential selection bias could limit the interpretation of our results. Thirdly, the cross-sectional design prevented us from determining the sequence of onset of seizures and cognitive impairment; consequently, we were unable to explain the causal relationship between EEG hyperexcitability and cognitive impairment in our cohort. Fourthly, the EEG features were analysed and annotated according to the SCORE system, and we have not completed further quantitative assessment. Future studies should clarify this discrepancy by investigating the potential of non-invasive EEG for risk stratification and early diagnosis of cognitive impairment in LOE, and by combining multimodal data to develop discrimination and prediction models with improved accuracy profiles.

## Conclusion

In summary, we described the EEG characteristics of LOE and highlighted a significant correlation between neurophysiological evidence of network disruption and cognitive impairment in the context of LOE. Scalp EEG, a non-invasive, low-cost, and highly accessible examination, could be a promising tool for predicting the development of cognitive impairment in LOE. Further exploration with expanded sample sizes or quantitative metrics is needed.

## Supplementary Material

fcag067_Supplementary_Data

## Data Availability

The data that support the findings of this study are not openly available due to privacy considerations, but are available from the corresponding author upon reasonable request. The codes generated and used in this work are available in the Supplementary Materials.
