# Supplementary material for: EEG features in late-onset epilepsy: possible correlation with cognitive impairment
Source: Brain Commun. 2026 Mar 14;8(2):fcag067. doi: 10.1093/braincomms/fcag067 (PMC13012000; doi:10.1093/braincomms/fcag067)
Supplement: fcag067_Supplementary_Data [file fcag067_supplementary_data.pdf]

Supplementary Table 1 Detailed EEG characteristics in the LOEU and LOED groups

	LOEU (n=177)	LOED (n=110)	P^a
Overall pathological, n (%)	128 (72)	95 (86)	**0.006***
**Background activity**			
Posterior dominant rhythm			
Frequency, median (IQR) (hz)	9.5 (1.0)	9.5 (1.0)	
Poor frequency regulation	9	7	
Amplitude (μV)			
< 20, n (%)	43	18	
20 ~ 50, n (%)	54	37	
50 ~ 100, n (%)	75	51	
100 ~ 200, n (%)	4	3	
> 200, n (%)	1	1	
Poor amplitude modulation, n (%)	9	7	
Reduced reactivity to eye-opening, n (%)	3	2	
Asymmetry, n (%)	2	4	
Amplitude asymmetry, n (%)	1	1	
Frequency asymmetry, n (%)	1	4	0.073
Absence, n (%)	0	0	
**Procedures**			
Intermittent photic stimulation			
No response n (%)	173	110	
Normal photic driving response, n (%)	4	0	
Hyperventilation			
Normal, n (%)	171	110	
Generalised slow waves, n (%)	3	0	
Epileptic activity, n (%)	3	0	
**Interictal findings**			
Slowing on EEG, n (%)	98 (55)	82 (75)	**0.001***
Generalised slowing, n (%)	17 (10)	19 (17)	0.067
Theta, n (%)	5 (3)	7 (6)	
Delta, n (%)	14 (8)	16 (15)	0.078
Focal slowing, n (%)	91 (51)	69 (63)	0.067
**Lateralization**			
Left, n (%)	40 (23)	35 (32)	0.098
Right, n (%)	26 (15)	28 (25)	**0.029***
Bilateral, n (%)	1 (1)	0	
Left and right, n (%)	24 (14)	6 (5)	**0.030***
**Frequency**			
Theta, n (%)	31 (17)	20 (18)	
Delta, n (%)	80 (45)	60 (55)	
**Location**			
Left temporal, n (%)	63 (36)	39 (35)	
Theta, n (%)	19 (11)	10 (9)	

Delta, n (%)	56 (32)	32 (29)	
Right temporal, n (%)	50 (28)	30 (27)	
Theta, n (%)	18 (10)	8 (7)	
Delta, n (%)	42 (24)	28 (25)	
Left and right temporal slowing, n (%)	24 (14)	6 (5)	**0.030***
Both theta, n (%)	3 (2)	1 (1)	
Delta on either hemisphere, n (%)	21 (12)	5 (5)	**0.036***
TIRDA, n (%)	48 (28)	30 (28)	
Left, n (%)	26 (15)	14 (13)	
Right, n (%)	17 (10)	16 (15)	
Left and right, n (%)	5 (3)	0	
IED on EEG, n (%)	99 (56)	63 (57)	
**Lateralization**			
Left, n (%)	34 (19)	29 (26)	
Right, n (%)	19 (11)	20 (18)	0.079
Bilateral, n (%)	5 (3)	3 (3)	
Left and right, n (%)	41 (23)	11 (10)	**0.007***
**Location**			
Left temporal, n (%)	33 (19)	25 (23)	
Right temporal, n (%)	18 (10)	18 (16)	
Left and right temporal, n (%)	40 (23)	11 (10)	**0.007***
Extra-temporal, n (%)	20 (11)	17 (15)	
**Location maximum**			
FT9/10, n (%)	59 (33)	28 (25)	
T3/T4, n (%)	27 (15)	15 (16)	
F7/8, n (%)	18 (10)	16 (15)	
T5/T6, n (%)	5 (3)	4 (4)	

[a]Only *P* values < 0.1 are shown. p-values showing statistical significance (< 0.05) are bolded and followed by asterisks (*).

Supplementary Table 2 Medication and cognitive impairment in LOE

	LOE-CI (n=140)	LOE-CN (n=147)	*P*[a]
**Current ASM therapy**			
Treatment naive, n (%)	54 (39)	70 (48)	
Currently not under treatment, n (%)	63 (45)	74 (50)	
Monotherapy	45 (32)	51 (35)	
Polytherapy	32 (23)	22 (15)	0.098
2, n (%)	29 (21)	17 (12)	
3, n (%)	2 (1)	5 (3)	
4, n (%)	1 (1)	0	
**Previous ASM therapy**			
# of ASMs trialed, median	0 (0)	0 (0)	
**Types of ASMs being used**			
Levetiracetam, n (%)	46 (33)	41 (28)	
Valproate, n (%)	29 (21)	26 (18)	
Oxcarbamazepine, n (%)	21 (15)	12 (8)	
Lacosamide, n (%)	8 (6)	5 (3)	
Perampanel, n (%)	3 (2)	5 (3)	
Lamotrigene, n (%)	2 (1)	3 (2)	
Clonazepam, n (%)	1 (1)	3 (2)	
Carbamazepine, n (%)	1 (1)	2 (1)	
Zonisamide, n (%)	0	2 (1)	
Topiramate, n (%)	0	2 (1)	
Pregabalin, n (%)	1 (1)	0	
**Concurrent Medication**			
Sleep aids, n (%)	13 (9)	17 (12)	
Antidepressants, n (%)	16 (11)	11 (7)	
Antipsychotics, n (%)	5 (4)	1 (1)	
Benzodiazepams, n (%)	2 (1)	5 (3)	
Memantine, n (%)	7 (5)	0	< 0.001
Donepezil, n (%)	14 (10)	0	< 0.001

[a]Only p-values < 0.1 are shown. *P* values showing statistical significance (< 0.05) are bolded and followed by asterisks (*)

Supplementary Table 3 Ordinal logistic regression

	LOE[a]			LOEU[a]		
	***P* value[b]**	**OR**	**95%CI**	***P* value[b]**	**OR**	**95%CI**
**Demographics**						
Age	**0.025***	1.035	1.008-1.062	**0.008***	1.048	1.016-1.080
Female	0.290	0.793	0.553-1.137	0.441	0.777	0.510-1.185
Years of education	0.257	0.971	0.930-1.013	0.120	0.952	0.905-1.000
**Personal History**						
Obesity	0.576	1.248	0.680-2.292	0.856	1.104	0.532-2.291
Smoking	0.857	1.040	0.680-1.589	0.676	0.840	0.501-1.406
Regular alcohol consumption	0.636	1.153	0.720-1.847	0.459	0.739	0.408-1.337
**Comorbidity**						
High blood pressure	0.466	1.180	0.819-1.700	0.840	1.079	0.696-1.673
Diabetes mellitus	0.621	1.171	0.719-1.908	0.856	1.072	0.601-1.915
Hyperlipidemia	0.370	1.336	0.789-2.261	0.877	1.051	0.559-1.974
Coronary heart disease	0.761	1.146	0.557-2.358	0.757	1.226	0.542-2.773
Mood disorder	**0.001**	3.427	1.826-6.430	**0.001***	4.518	2.094-9.750
Sleep disorder	**0.027***	1.743	1.113-2.681	**0.005***	2.292	1.387-3.788
**Epilepsy characteristics**						
Age at seizure onset	-	-	-	-	-	-
Epilepsy Duration	-	-	-	-	-	-
Active seizure during the previous 6 months	-	-	-	-	-	-
Number of ASMs	-	-	-	-	-	-
History of BTCS	-	-	-	-	-	-
**Medical History**						
History of stroke	**0.001***	3.428	1.764-6.660	-	-	-
History of head injury	0.098	2.362	0.983-5.676	-	-	-
History of cerebral infection	**0.036***	6.802	1.395-33.178	-	-	-
History of cerebral surgery	**0.001***	4.145	1.965-8.744	-	-	-
Brain tumor	**0.031***	3.074	1.257-7.519	-	-	-
**MRI**						
Overall pathological finding	**<0.001***	2.461	1.638-3.699	0.293	1.414	0.888-2.250
Generalised cerebral atrophy	**0.016***	1.787	1.191-2.681	0.127	1.668	0.998-2.787
Hippocampal sclerosis	0.145	2.393	0.885-6.476	0.856	1.304	0.181-9.404
CSVD sign	0.166	1.378	0.933-2.036	0.757	0.878	0.534-1.442
Vascular malformation	0.857	0.929	0.419-2.062	0.867	0.883	0.254-3.073
Encephalomalacia	**0.001***	2.642	1.602-4.357	-	-	-
**EEG**						
Overall abnormal EEG	**<0.001***	8.416	5.517-12.838	**<0.001***	6.828	4.271-10.917
Slowing on EEG	**<0.001***	5.029	3.424-7.385	**<0.001***	4.102	2.632-6.393
**Generalised slowing**						
Theta	0.756	0.840	0.357-1.978	0.448	0.534	0.172-1.663
Delta	0.092	1.877	1.002-3.517	0.457	1.545	0.683-3.495
**Focal slowing**						

**Left temporal**						
Theta	0.857	0.920	0.419-2.022	0.770	0.797	0.295-2.153
Delta	**<0.001***	3.068	1.994-4.721	**<0.001***	3.685	2.204-6.161
**Right temporal**						
Theta	0.709	1.267	0.482-3.331	0.459	1.702	0.596-4.864
Delta	**<0.001***	4.759	2.884-7.853	**<0.001***	5.854	3.171-10.805
**Bilateral temporal**						
Both theta	0.290	0.432	0.117-1.591	0.670	0.595	0.138-2.558
Delta on either hemisphere	**0.025***	2.553	1.259-5.179	**0.006***	3.381	1.572-7.271
**TIRDA**	**<0.001***	7.950	4.709-13.423	**<0.001***	9.815	5.211-18.489
Left	**<0.001***	6.732	3.451-13.134	**<0.001***	8.345	3.791-18.370
Right	**<0.001***	9.257	4.211-20.348	**<0.001***	10.646	3.901-29.049
Left and right	**0.039***	13.457	1.515-119.560	**0.017***	21.485	2.398-192.512
**IED on EEG**						
**Anterior temporal IED**						
FT9	**<0.001***	6.627	3.188-13.779	**<0.001***	10.292	4.219-25.105
FT10	**<0.001***	9.866	3.778-25.762	**<0.001***	20.225	5.237-78.100
FT9 and FT10	**<0.001***	22.441	8.453-59.576	**<0.001***	43.602	14.311-132.842
**Other temporal IEDs**						
Left	**0.036***	2.086	1.136-3.829	0.322	1.724	0.795-3.739
Right	**0.025***	2.657	1.262-5.593	0.181	2.336	0.889-6.134
Left and right	0.393	1.486	0.695-3.175	0.159	2.142	0.939-4.886
**Extratemporal IEDs**						
Left	0.117	2.880	0.925-8.971	0.448	2.192	0.535-8.983
Right	0.347	1.903	0.628-5.769	0.715	1.567	0.354-6.944
Left and right	0.162	2.880	0.812-10.220	0.448	2.192	0.535-8.983

[a]To perform ordinal logistic regression, the outcome variable was adapted into an ordered factor: LOE (HC < LOE-CN < LOE-CI), LOEU (HC < LOEU-CN < LOEU-CI).

[b]*P* values presented below have been adjusted for multiple comparisons using FDR correction. *P* values showing statistical significance (< 0.05) are bolded and followed by asterisks (*).

Supplementary Table 4 Binomial logistic regression between LOE-CI and LOE-CN

	Univariate			Multivariate[a]		
	***P* value[b]**	**OR**	**95%CI**	***P* value[b]**	**OR**	**95%CI**
**Demographics**						
Age	0.195	1.034	1.000-1.069	-	-	-
Female	0.769	1.114	0.692-1.795	-	-	-
Years of education	0.442	0.964	0.912-1.020	-	-	-
**Personal History**						
Obesity	0.896	1.063	0.485-2.331	0.808	1.135	0.512-2.514
Smoking	0.539	1.356	0.776-2.370	0.394	1.534	0.831-2.832
Regular alcohol consumption	0.167	1.916	1.025-3.582	0.108	2.148	1.106-4.174
**Comorbidity**						
High blood pressure	0.762	0.879	0.546-1.417	0.674	0.818	0.496-1.349
Diabetes mellitus	0.655	1.304	0.693-2.456	0.589	1.413	0.739-2.700
Hyperlipidemia	0.484	1.503	0.766-2.952	0.419	1.563	0.790-3.095
Coronary heart disease	0.791	1.179	0.464-2.995	0.976	0.985	0.374-2.594
Mood disorder	0.167	2.133	1.039-4.378	0.218	2.072	0.976-4.398
Sleep disorder	0.769	0.885	0.517-1.514	0.674	0.825	0.473-1.440
**Epilepsy characteristics**						
Age at seizure onset	0.331	1.027	0.993-1.061	0.674	0.965	0.876-1.064
Epilepsy Duration	0.518	1.005	0.997-1.013	0.674	1.003	0.995-1.011
Active seizure during the previous 6 months	0.684	1.205	0.726-1.998	0.674	1.183	0.705-1.985
Number of ASMs	0.442	1.200	0.908-1.585	0.300	1.255	0.944-1.669
History of BTCS	0.684	1.186	0.742-1.895	0.674	1.173	0.729-1.886
**Medical History**						
History of stroke	0.684	1.298	0.627-2.689	0.605	1.452	0.689-3.060
History of head injury	0.770	0.807	0.292-2.229	0.808	0.851	0.304-2.378
History of cerebral infection	0.395	3.246	0.644-16.362	0.263	4.199	0.814-21.646
History of cerebral surgery	0.437	1.709	0.770-3.790	0.270	1.987	0.881-4.480
Brain tumor	0.791	1.194	0.447-3.186	0.808	1.203	0.436-3.318
**MRI**						
Overall pathological finding	0.274	1.642	0.926-2.912	0.309	1.577	0.879-2.829
Generalised cerebral atrophy	0.609	1.278	0.778-2.100	0.808	1.124	0.645-1.956
Hippocampal sclerosis	0.846	1.148	0.388-3.394	0.808	1.194	0.401-3.555
CSVD signs	0.195	1.688	0.987-2.887	0.251	1.651	0.957-2.846
Vascular malformation	0.655	0.625	0.219-1.788	0.589	0.574	0.198-1.660
Encephalomalacia	0.762	1.176	0.648-2.135	0.674	1.228	0.672-2.244
**EEG**						
Overall abnormal EEG	**< 0.001***	6.612	3.278-13.338	**< 0.001***	6.291	3.099-12.772
Slowing on EEG	**< 0.001***	3.516	2.111-5.855	**< 0.001***	3.317	1.974-5.574
**Generalised slowing**						
Theta	0.766	0.741	0.230-2.391	0.674	0.592	0.178-1.967
Delta	0.083	2.706	1.194-6.134	0.154	2.415	1.052-5.541
**Focal slowing**						

**Left temporal**						
Theta	0.762	0.729	0.235-2.260	0.795	0.765	0.244-2.394
Delta	**0.020***	2.187	1.300-3.681	**0.039***	2.072	1.222-3.511
**Right temporal**						
Theta	0.769	1.326	0.372-4.720	0.808	1.259	0.349-4.541
Delta	**0.010***	2.541	1.444-4.471	**0.012***	2.514	1.419-4.454
**Bilateral temporal**						
Both theta	0.655	0.369	0.038-3.593	0.646	0.344	0.035-3.425
Delta on either hemisphere	0.274	2.090	0.899-4.862	0.270	2.050	0.871-4.823
**TIRDA**	**0.001***	3.218	1.849-5.601	**0.001***	3.138	1.796-5.484
Left	**0.036***	2.656	1.311-5.380	0.052	2.546	1.248-5.197
Right	**0.010***	3.814	1.690-8.609	**0.010***	3.915	1.722-8.901
Left and right	0.331	5.721	0.628-52.077	0.419	4.455	0.474-41.843
**IED on EEG**						
**Anterior temporal IEDs**						
FT9	**0.001***	6.509	2.670-15.867	**0.001**	6.203	2.531-15.200
FT10	**0.014***	4.746	1.763-12.782	**0.014**	4.780	1.759-12.986
FT9 and FT10	**< 0.001***	20.251	5.988-68.489	**< 0.001***	20.659	6.046-70.587
**Other temporal IEDs**						
Left	0.655	0.730	0.348-1.531	0.674	0.761	0.359-1.612
Right	0.762	1.288	0.541-3.065	0.674	1.322	0.550-3.180
Left and right	0.429	0.495	0.179-1.368	0.263	0.401	0.142-1.137
**Extratemporal IEDs**						
Left	0.684	1.595	0.440-5.785	0.674	1.628	0.446-5.942
Right	0.846	0.851	0.223-3.240	0.904	0.889	0.228-3.471
Left and right	0.932	1.063	0.260-4.343	0.932	1.085	0.256-4.603

[a]Age, sex, and years of education are included as covariates in the multivariate analysis.

[b]*P* values presented below have been adjusted for multiple comparisons using FDR correction. *P* values showing statistical significance (< 0.05) are bolded and followed by asterisks (*).

Supplementary Table 5 Binomial logistic regression between LOEU-CI and LOEU-CN

	Univariate			Multivariate[a]		
	P value[b]	OR	95%CI	*P* value[b]	OR	95%CI
**Demographics**						
Age	**0.034***	1.062	1.017-1.109	-	-	-
Female	0.892	1.100	0.598-2.021	-	-	-
Years of education	0.235	0.939	0.873-1.010	-	-	-
**Personal History**						
Obesity	0.892	0.788	0.273-2.272	0.955	0.910	0.309-2.681
Smoking	0.710	1.383	0.654-2.928	0.597	1.572	0.689-3.590
Regular alcohol consumption	0.392	1.828	0.755-4.427	0.315	2.137	0.824-5.543
**Comorbidity**						
High blood pressure	0.892	0.893	0.476-1.676	0.726	0.744	0.375-1.474
Diabetes mellitus	0.892	1.248	0.549-2.837	0.676	1.504	0.636-3.558
Hyperlipidemia	0.892	1.095	0.446-2.685	0.927	1.248	0.494-3.149
Coronary heart disease	0.892	1.123	0.361-3.487	0.927	0.746	0.220-2.527
Mood disorder	0.112	2.782	1.113-6.957	0.176	2.672	1.005-7.103
Sleep disorder	0.915	0.964	0.496-1.875	0.944	0.908	0.443-1.857
**Epilepsy characteristics**						
Age at seizure onset	0.085	1.053	1.008-1.100	0.836	0.951	0.829-1.091
Epilepsy Duration	0.389	1.008	0.997-1.019	0.854	1.004	0.992-1.016
Active seizure during the previous 6 months	0.892	0.939	0.483-1.823	0.944	0.924	0.461-1.853
Number of ASMs	0.892	0.915	0.609-1.375	0.944	0.955	0.626-1.458
History of BTCS	0.892	1.067	0.583-1.952	0.997	0.994	0.530-1.861
**Medical History**						
History of stroke	-	-	-	-	-	-
History of head injury	-	-	-	-	-	-
History of cerebral infection	-	-	-	-	-	-
History of cerebral surgery	-	-	-	-	-	-
Brain tumor	-	-	-	-	-	-
**MRI**						
Overall pathological finding	0.710	1.347	0.666-2.728	0.931	1.183	0.555-2.521
Generalised cerebral atrophy	0.831	1.246	0.623-2.494	0.997	1.003	0.473-2.128
Hippocampal sclerosis	0.892	0.753	0.046-12.408	0.997	0.994	0.056-17.548
CSVD signs	0.307	1.783	0.847-3.749	0.541	1.585	0.732-3.430
Vascular malformation	0.892	1.442	0.236-8.805	0.997	0.928	0.137-6.286
Encephalomalacia	-	-	-	-	-	-
**EEG**						
Overall abnormal EEG	**< 0.001***	8.927	3.548-22.457	**0.001***	7.837	3.073-19.983
Slowing on EEG	**0.001***	3.683	1.942-6.982	**0.007***	3.216	1.665-6.212
**Generalised slowing**						
Theta	0.578	0.316	0.035-2.884	0.368	0.183	0.019-1.816
Delta	0.058	5.389	1.448-20.060	0.175	4.056	1.053-15.616
**Focal slowing**						

**Left temporal**						
Theta	0.892	0.712	0.132-3.836	0.944	0.736	0.134-4.052
Delta	**0.019***	2.752	1.424-5.317	**0.042***	2.654	1.334-5.277
**Right temporal**						
Theta	0.752	1.702	0.407-7.127	0.927	1.501	0.346-6.515
Delta	**0.034***	2.766	1.347-5.679	0.085	2.527	1.196-5.342
**Bilateral temporal**						
Both theta	0.892	0.714	0.063-8.049	0.933	0.601	0.050-7.221
Delta on either hemisphere	0.225	2.321	0.909-5.929	0.313	2.221	0.840-5.875
**TIRDA**	**0.019***	2.908	1.465-5.772	**0.043***	2.657	1.314-5.374
Left	0.147	2.379	1.010-5.604	0.267	2.189	0.909-5.275
Right	0.113	3.199	1.111-9.208	0.175	3.108	1.048-9.221
Left and right	0.233	6.979	0.758-64.287	0.407	4.846	0.502-46.827
**IED on EEG**						
**Anterior temporal IEDs**						
FT9	**< 0.001***	13.481	4.156-43.736	**0.001***	13.190	3.933-44.240
FT10	**0.019***	8.988	2.228-36.255	**0.026***	9.237	2.162-39.462
FT9 and FT10	**< 0.001***	43.815	9.766-196.564	**< 0.001***	53.280	11.359-249.917
**Other temporal IEDs**						
Left	0.133	0.256	0.070-0.939	0.175	0.255	0.067-0.965
Right	0.915	0.924	0.269-3.175	0.944	0.840	0.231-3.057
Left and right	0.578	0.555	0.180-1.709	0.313	0.380	0.116-1.244
**Extratemporal IEDs**						
Left	0.892	0.822	0.134-5.051	0.944	0.804	0.125-5.181
Right	0.744	0.411	0.042-4.035	0.927	0.546	0.053-5.564
Left and right	0.578	0.308	0.034-2.818	0.664	0.314	0.030-3.241

[a]Age, sex, and years of education are included as covariates in the multivariate analysis.

[b]*P* values presented below have been adjusted for multiple comparisons using FDR correction. *P* values showing statistical significance (< 0.05) are bolded and followed by asterisks (*).

Supplementary Table 6 Binomial logistic regression between LOED-CI and LOED-CN

	Univariate			Multivariate[a]		
	P value[b]	OR	95% CI	*P* value[b]	OR	95% CI
**Demographics**						
Age	0.943	0.993	0.939-1.050	-	-	-
Female	0.928	1.192	0.542-2.625	-	-	-
Years of education	0.943	0.986	0.900-1.082	-	-	-
**Personal History**						
Obesity	0.910	1.695	0.477-6.018	0.780	1.666	0.461-6.014
Smoking	0.928	1.167	0.497-2.740	0.780	1.361	0.521-3.552
Regular alcohol consumption	0.864	1.689	0.680-4.193	0.694	1.967	0.744-5.201
**Comorbidity**						
High blood pressure	0.910	0.733	0.343-1.569	0.780	0.755	0.345-1.654
Diabetes mellitus	0.928	1.345	0.485-3.728	0.780	1.404	0.499-3.949
Hyperlipidemia	0.776	2.184	0.720-6.627	0.694	2.137	0.697-6.548
Coronary heart disease	0.928	1.525	0.267-8.701	0.813	1.681	0.281-10.059
Mood disorder	0.928	1.400	0.437-4.489	0.857	1.347	0.400-4.530
Sleep disorder	0.943	0.871	0.340-2.229	0.922	0.864	0.333-2.245
**Epilepsy characteristics**						
Age at seizure onset	0.943	0.995	0.943-1.051	0.982	1.011	0.877-1.167
Epilepsy Duration	0.943	0.999	0.987-1.011	0.986	0.999	0.987-1.011
Active seizure during the previous 6 months	0.734	1.852	0.836-4.104	0.669	1.880	0.839-4.210
Number of ASMs	0.776	1.368	0.887-2.110	0.694	1.385	0.891-2.154
History of BTCS	0.910	1.340	0.624-2.876	0.780	1.345	0.624-2.900
**Medical History**						
History of stroke	0.928	0.853	0.375-1.941	0.922	0.877	0.374-2.056
History of head injury	0.910	0.609	0.204-1.819	0.780	0.618	0.205-1.867
History of cerebral infection	0.864	2.368	0.456-12.300	0.780	2.446	0.457-13.097
History of cerebral surgery	0.928	1.209	0.504-2.901	0.921	1.176	0.477-2.897
Brain tumor	0.928	0.813	0.288-2.293	0.780	0.697	0.226-2.147
**MRI**						
Overall pathological finding	0.928	2.105	0.188-23.575	0.780	2.483	0.206-29.979
Generalised cerebral atrophy	0.928	1.212	0.540-2.720	0.780	1.377	0.543-3.493
Hippocampal sclerosis	0.943	1.148	0.314-4.200	0.955	1.156	0.314-4.256
CSVD sign	0.928	1.198	0.538-2.670	0.886	1.191	0.523-2.711
Vascular malformation	0.631	0.281	0.068-1.150	0.640	0.281	0.068-1.155
Encephalomalacia	0.864	0.652	0.288-1.476	0.780	0.654	0.284-1.506
**EEG**						
Overall abnormal EEG	0.541	3.135	0.993-9.902	0.545	3.156	0.975-10.219
Slowing on EEG	0.511	2.679	1.109-6.468	0.487	2.868	1.115-7.379
**Generalised slowing**						
Theta	0.994	0.994	0.212-4.671	0.991	1.011	0.208-4.920
Delta	0.928	1.289	0.433-3.839	0.857	1.304	0.433-3.924
**Focal slowing**						

**Left temporal**						
Theta	0.928	0.615	0.128-2.953	0.780	0.541	0.108-2.703
Delta	0.864	1.566	0.659-3.726	0.780	1.593	0.658-3.854
**Right temporal**						
Theta	0.994	0.905	0.055-14.974	0.991	0.935	0.055-16.022
Delta	0.631	2.262	0.892-5.733	0.660	2.242	0.868-5.788
**Bilateral temporal**						
Both theta	0.994	-	Inf	0.991	-	Inf
Delta on either hemisphere	0.864	3.051	0.330-28.243	0.780	2.858	0.299-27.289
**TIRDA**[c]	0.282	4.205	1.553-11.388	**0.240**	4.330	1.587-11.818
Left	0.541	3.855	0.999-14.866	**0.526**	4.137	1.052-16.273
Right	0.511	4.556	1.205-17.220	**0.487**	4.519	1.180-17.300
Left and right	-	-	-	-	-	-
**IED on EEG**						
**Anterior temporal IEDs**						
FT9	0.806	2.540	0.629-10.256	0.694	2.555	0.630-10.362
FT10	0.864	2.222	0.537-9.195	0.780	2.158	0.518-8.994
FT9 and FT10	0.731	5.714	0.658-49.593	0.669	5.744	0.659-50.079
**Other temporal IEDs**						
Left	0.910	1.492	0.502-4.439	0.780	1.507	0.504-4.503
Right	0.910	1.628	0.452-5.858	0.780	1.591	0.437-5.792
Left and right	0.910	0.407	0.035-4.677	0.780	0.422	0.036-5.005
**Extratemporal IEDs**						
Left	0.864	3.321	0.358-30.806	0.780	3.432	0.361-32.616
Right	0.943	1.245	0.199-7.789	0.955	1.220	0.192-7.764
Left and right	0.994	-	Inf	0.991	-	Inf

[a]Age, sex, and years of education are included as covariates in the multivariate analysis.

[b]*P* values presented below have been adjusted for multiple comparisons using FDR correction. *P* values showing statistical significance (< 0.05) are bolded and followed by asterisks (*).

[c]Unadjusted *P* values for TIRDA variables were: TIRDA (*P*=0.005), left TIRDA (*P*=0.045), right TIRDA (*P*=0.030). These results are presented because TIRDA was the only significant variable before correction for multiple comparisons, and the extent of significance appeared to be largely restricted by the correction. The corresponding *P* values are bolded and followed by asterisks (*).

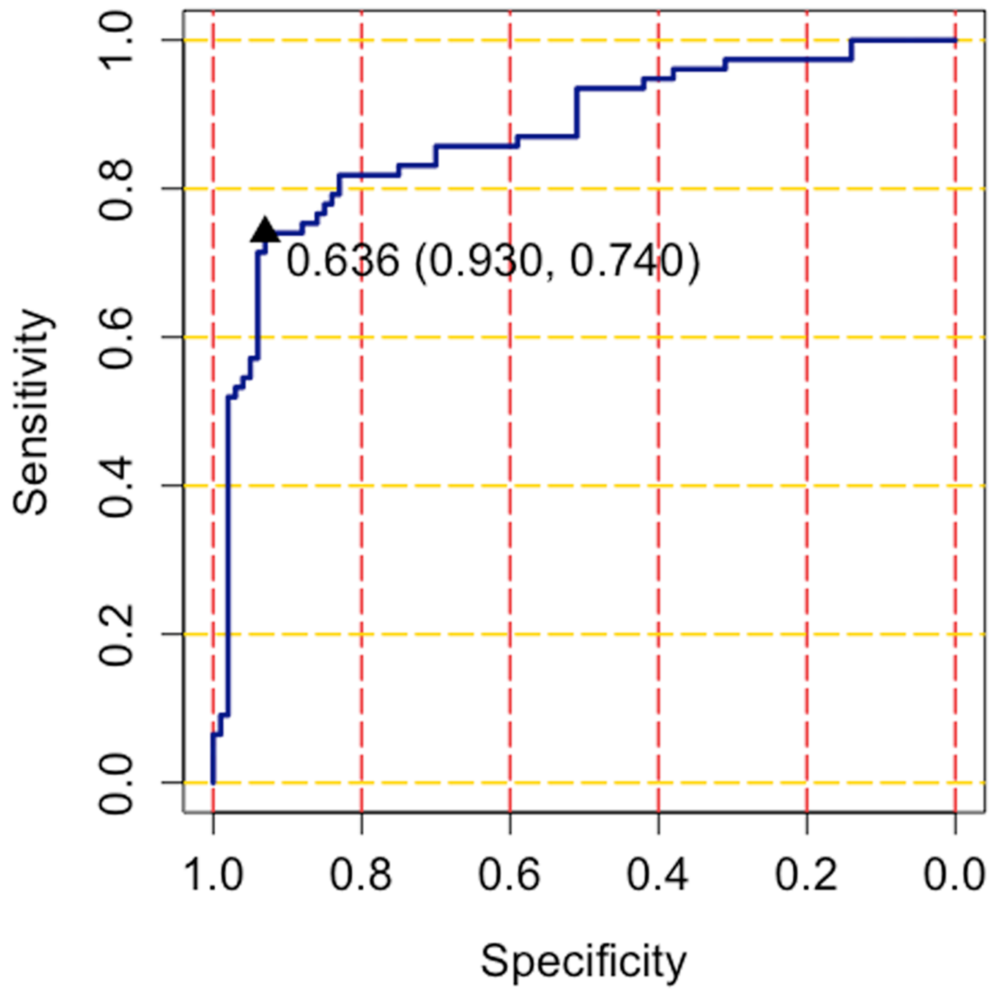

Supplementary Fig. 1 LASSO model performance Receiver operating characteristic curve analysis was performed with LOEU-CI (n=76) vs. LOEU-CN (n=101). AUROC = 0.869 (95% CI, 0.813-0.925), sensitivity = 0.891 (95% CI, 0.788-0.955), specificity = 0.823 (95% CI, 0.740-0.888), positive predictive value = 0.740 (95% CI, 0.628-0.834), negative positive value = 0.930 (95% CI, 0.861-0.971), accuracy = 0.847 (95% CI, 0.786-0.897). AUROC = area under the receiver operating characteristic curve; LASSO = least absolute shrinkage and selection operator; LOEU-CN = late-onset epilepsy of unknown aetiology with normal cognition; LOEU-CI = late-onset epilepsy of unknown aetiology with cognitive impairment.

Supplementary Material
Codes for analyses in this study

```
######### This code is   for use in R studio ############
######### Download and Install necessary packages ############
######### Data Preprocessing and formatting ############
data <- LOE_EEG[,-1]
data <- data[,-1]
# Converting Numerical Data
data$BMI <- as.numeric(data$BMI)
data$CurrASM<- as.numeric(data$CurrASM)
data$FailASM <- as.numeric(data$FailASM)
data$Edu_yrs <- as.numeric(data$Edu_yrs)
data$Age <- as.numeric(data$Age)
data$Onset_year <- as.numeric(data$Onset_year)
# Converting Non-ordinal Categorical Data
data$Sex <- factor(data$Sex, labels = c("Male", "Female"))
data$Sex <- relevel(data$Sex, ref = "Male")
data$FS.theta <- factor(data$FS.theta, levels = c("0", "Left", "Right", "Left +
Right"), ordered = FALSE)
data$FS.theta <- relevel(data$FS.theta, ref = "0")
data$FS.delta <- factor(data$FS.delta, levels = c("0", "Left", "Right", "Left +
Right"), ordered = FALSE)
data$FS.delta <- relevel(data$FS.delta, ref = "0")
data$FS.bilateral <- factor(data$FS.bilateral, levels = c("0", "Theta", "Delta"),
ordered = FALSE)
data$FS.bilateral <- relevel(data$FS.bilateral, ref = "0")
data$Ant_Tem_IED <- factor(data$Ant_Tem_IED, levels = c("0", "FT9",
"FT10", "FT9 + FT10"), ordered = FALSE)
data$Ant_Tem_IED <- relevel(data$Ant_Tem_IED, ref = "0")
data$Oth_Tem_IED <- factor(data$Oth_Tem_IED, levels = c("0", "Left",
"Right", "Left + Right"), ordered = FALSE)
data$Oth_Tem_IED <- relevel(data$Oth_Tem_IED, ref = "0")
data$Extra_Tem_IED <- factor(data$Extra_Tem_IED, levels = c("0", "Left",
"Right", "Left + Right"), ordered = FALSE)
data$Extra_Tem_IED <- relevel(data$Extra_Tem_IED, ref = "0")
data$FS.left <- factor(data$FS.left, levels = c("0", "theta", "delta"), ordered =
FALSE)
data$FS.left <- relevel(data$FS.left, ref = "0")
data$FS.right <- factor(data$FS.right, levels = c("0", "theta", "delta"), ordered
= FALSE)
data$FS.right <- relevel(data$FS.right, ref = "0")
data$TIRDA <- factor(data$TIRDA, levels = c("0", "Left", "Right", "Left +
Right"), ordered = FALSE)
```

```
data$TIRDA <- relevel(data$TIRDA, ref = "0")
# Converting Ordinal Categorical Data
data$Edu <- factor(data$Edu, levels = c("文盲", "小学", "初中", "高中", "大学"),
ordered = TRUE)
levels(data$Edu)
data$SzF <- factor(data$SzF, levels = c("2-year seizure-free", "1-year
seizure-free", "yearly", "seasonly", "monthly", "weekly", "daily"), ordered =
TRUE)
levels(data$SzF)
# Converting Binomial Data
data <- data %>%
  mutate(across(where(~ all(na.omit(.x) %in% c(0, 1))), ~ factor(.x, levels =
c(0, 1))))

######### HC vs. LOE-CN vs. LOE-CI Ordinal Regression ############
# Defining Ordinal Outcome
data_LOE_Ordinal <- data
data_LOE_Ordinal$Outcome_LOE <-
factor(data_LOE_Ordinal$Outcome_LOE, levels = c("HC", "LOE-CN", "LOE-
CI"), ordered = TRUE)
######### MICE Multiple Imputation
# Selecting the variables to be included in MICE dataset
Variables_overall <- c("Age", "Sex", "Edu_yrs", "Overweight", "BMI",
"Obesity", "Onset_year", "Active_Sz", "EpiDur", "BTCS", "Sleep.disorder",
"Mood.disorder", "HBP", "DM", "Lipidemia", "CHD", "Smoking", "Stroke",
"Trauma", "Surgery", "Cer_Infection", "Tumor", "Alcohol_Consume",
"Image_Pos", "Cerebral_Atrophy", "HS", "SVD", "Vascular_Mal", "Malacia",
"CurrMed", "EEG_Con", "SWA", "G.theta", "G.delta", "FS.left", "FS.right",
"FS.bilateral", "TIRDA", "TIRDA_total", "Ant_Tem_IED", "Oht_Tem_IED",
"Extra_Tem_IED")
formula <- as.formula(paste("Outcome_LOE ~", paste(Variables_overall,
collapse = " + ")))
data_LOE_Ordinal <- data_LOE_Ordinal[, c("Outcome_LOE",
Variables_overall)]
# Defining imputation methods
md.pattern(data_LOE_Ordinal)
vars_with_missing <-
names(data_LOE_Ordinal)[colSums(is.na(data_LOE_Ordinal)) > 0]
vars_without_missing <- setdiff(names(data_LOE_Ordinal),
vars_with_missing)
methods <- make.method(data_LOE_Ordinal)
methods["BMI"] <- "pmm"
methods["Obesity"] <- "logreg"
```

```
methods["Edu_yrs"] <- "pmm"
methods["Active_Sz"] <- "logreg"
methods["Image_Pos"] <- "logreg"
methods["Cerebral_Atrophy"] <- "logreg"
methods["HS"] <- "logreg"
methods["SVD"] <- "logreg"
methods["Vascular_Mal"] <- "logreg"
methods["Malacia"] <- "logreg"
pred_matrix <- make.predictorMatrix(data_LOE_Ordinal)
pred_matrix[, vars_without_missing] <- 0
# MICE
imp <- mice(data_LOE_Ordinal, m =10, method = methods, seed = 1234,
predictorMatrix = pred_matrix)
summary(imp)
######### Ordinal Logistic Regression and FDR correction
exploratory_vars <- c("Age", "Sex", "Edu_yrs", "Obesity", "HBP", "DM",
"Lipidemia", "CHD", "Smoking", "Alcohol_Consume", "Sleep.disorder",
"Mood.disorder", "Stroke", "Trauma", "Surgery", "Cer_Infection", "Tumor",
"Image_Pos", "Cerebral_Atrophy", "HS", "SVD", "Vascular_Mal", "Malacia",
"EEG_Con", "SWA", "G.theta", "G.delta", "FS.left", "FS.right", "FS.bilateral",
"TIRDA_total", "TIRDA", "Ant_Tem_IED", "Oht_Tem_IED", "Extra_Tem_IED")
outcome_name <- "Outcome_LOE"
conf_level <- 0.95
results_list <- list()
for (var in exploratory_vars) {
  formula_text <- paste0(outcome_name, " ~ `", var, "`")
  mm_names <- character(0)
  try({
    mm <- model.matrix(as.formula(formula_text), data = comp1)
    mm_names <- colnames(mm)
    mm_names <- setdiff(mm_names, "(Intercept)")
  }, silent = TRUE)
  res_obj <- tryCatch({
    call_text <- paste0("with(imp, clm(", formula_text, "))")
    fit <- eval(parse(text = call_text))
    pooled <- pool(fit)
    summary_pooled <- summary(pooled, conf.int = TRUE, conf.level =
conf_level)
    summary_pooled <- summary_pooled %>%
      dplyr::mutate(
        OR = exp(estimate),
        OR_low = exp(estimate - 1.96 * std.error),
        OR_high = exp(estimate + 1.96 * std.error)
      )
```

```
    matched_idx <- rep(FALSE, nrow(summary_pooled))
    if(length(mm_names) > 0) {
      matched_idx <- sapply(summary_pooled$term, function(t) {
        any(sapply(mm_names, function(m) {
          grepl(m, t, fixed = TRUE)
        }))
      })
    }
    if (!any(matched_idx)) {
      matched_idx <- grepl(var, summary_pooled$term, fixed = TRUE)
    }
    outcome_rows <- summary_pooled[matched_idx, , drop = FALSE]
    outcome_rows <- outcome_rows %>% mutate(Variable = var, model_call
= call_text)
    list(ok = TRUE, var = var, fit = fit, pooled = pooled,
         summary_pooled = summary_pooled, outcome_rows =
outcome_rows)
  }, error = function(e) {
    warning("Variable", var, " Failure： ", e$message)
    list(ok = FALSE, var = var, error = e$message, outcome_rows = NULL)
  })
  results_list[[var]] <- res_obj
}
all_outcomes <- do.call(rbind, lapply(results_list, function(x) {
  if(!is.null(x) && isTRUE(x$ok) && !is.null(x$outcome_rows) &&
nrow(x$outcome_rows) > 0) {
    df <- x$outcome_rows
    df %>% dplyr::select(Variable, term, estimate, std.error, statistic, df,
p.value, OR, OR_low, OR_high, model_call)
  } else {
    NULL
  }
}))
if (is.null(all_outcomes) || nrow(all_outcomes) == 0) {
  stop("Failed to match results")
}
all_outcomes <- all_outcomes %>%
  mutate(
    p_adj_BH = p.adjust(p.value, method = "BH"),
    p_adj_bonf = p.adjust(p.value, method = "bonferroni")
  )
all_outcomes <- all_outcomes %>%
  mutate(
```

```
    OR_fmt = ifelse(!is.na(OR), sprintf("%.3f", OR), NA_character_),
    CI_fmt = ifelse(!is.na(OR_low) & !is.na(OR_high),
                    paste0(sprintf("%.3f", OR_low), " - ", sprintf("%.3f",
OR_high)),
                    NA_character_),
    p_raw_fmt = ifelse(!is.na(p.value), sprintf("%.3f", p.value),
NA_character_),
    p_adj_BH_fmt = ifelse(!is.na(p_adj_BH), sprintf("%.3f", p_adj_BH),
NA_character_),
    p_adj_bonf_fmt = ifelse(!is.na(p_adj_bonf), sprintf("%.3f", p_adj_bonf),
NA_character_)
  ) %>%
  dplyr::select(Variable, term, OR_fmt, CI_fmt, p_raw_fmt, p_adj_BH_fmt,
p_adj_bonf_fmt, model_call)
# Saving results
write.csv(all_outcomes, "Univariate_ordinal_LOE.csv", row.names = FALSE)

######### HC vs. LOEU-CN vs. LOEU-CI Ordinal Regression
############
# Defining Ordinal Outcome
data_LOEU_Ordinal <- subset(data, Outcome != "nonLOEU-CN")
data_LOEU_Ordinal <- subset(data_LOEU_Ordinal, Outcome != "nonLOEU-
CI")
data_LOEU_Ordinal$Outcome <- factor(data_LOEU_Ordinal$Outcome,
levels = c("HC", "LOEU-CN", "LOEU-CI"), ordered = TRUE)
######### MICE Multiple Imputation
# Selecting the variables to be included in MICE dataset
Variables_overall <- c("Age", "Sex", "Edu_yrs", "Overweight", "BMI",
"Obesity", "Onset_year", "Active_Sz", "EpiDur", "BTCS", "Sleep.disorder",
"Mood.disorder", "HBP", "DM", "Lipidemia", "CHD", "Smoking", "Stroke",
"Trauma", "Surgery", "Cer_Infection", "Tumor", "Alcohol_Consume",
"Image_Pos", "Cerebral_Atrophy", "HS", "SVD", "Vascular_Mal", "Malacia",
"CurrMed", "EEG_Con", "SWA", "G.theta", "G.delta", "FS.left", "FS.right",
"FS.bilateral", "TIRDA", "TIRDA_total", "Ant_Tem_IED", "Oht_Tem_IED",
"Extra_Tem_IED")
formula <- as.formula(paste("Outcome ~", paste(Variables_overall, collapse =
" + ")))
data_LOEU_Ordinal <- data_LOEU_Ordinal[, c("Outcome",
Variables_overall)]
# Defining imputation methods
md.pattern(data_LOEU_Ordinal)
vars_with_missing <-
names(data_LOEU_Ordinal)[colSums(is.na(data_LOEU_Ordinal)) > 0]
vars_without_missing <- setdiff(names(data_LOEU_Ordinal),
```

```
vars_with_missing)
methods <- make.method(data_LOEU_Ordinal)
methods["BMI"] <- "pmm"
methods["Obesity"] <- "logreg"
methods["Edu_yrs"] <- "pmm"
methods["Active_Sz"] <- "logreg"
methods["Image_Pos"] <- "logreg"
methods["Cerebral_Atrophy"] <- "logreg"
methods["HS"] <- "logreg"
methods["SVD"] <- "logreg"
methods["Vascular_Mal"] <- "logreg"
methods["Malacia"] <- "logreg"
pred_matrix <- make.predictorMatrix(data_LOEU_Ordinal)
pred_matrix[, vars_without_missing] <- 0
# MICE
imp <- mice(data_LOEU_Ordinal, m =10, method = methods, seed = 1234,
predictorMatrix = pred_matrix)
summary(imp)
######### Ordinal Logistic Regression and FDR correction
exploratory_vars <- c("Age", "Sex", "Edu_yrs", "Obesity", "HBP", "DM",
"Lipidemia", "CHD", "Smoking", "Alcohol_Consume", "Sleep.disorder",
"Mood.disorder", "Image_Pos", "Cerebral_Atrophy", "HS", "SVD",
"Vascular_Mal", "EEG_Con", "SWA", "G.theta", "G.delta", "FS.left", "FS.right",
"FS.bilateral", "TIRDA_total", "TIRDA", "Ant_Tem_IED", "Oht_Tem_IED",
"Extra_Tem_IED")
outcome_name <- "Outcome"
conf_level <- 0.95
results_list <- list()
for (var in exploratory_vars) {
  formula_text <- paste0(outcome_name, " ~ `", var, "`")
  mm_names <- character(0)
  try({
    mm <- model.matrix(as.formula(formula_text), data = comp1)
    mm_names <- colnames(mm)
    mm_names <- setdiff(mm_names, "(Intercept)")
  }, silent = TRUE)
  res_obj <- tryCatch({
    call_text <- paste0("with(imp, clm(", formula_text, "))")
    fit <- eval(parse(text = call_text))
    pooled <- pool(fit)
    summary_pooled <- summary(pooled, conf.int = TRUE, conf.level =
conf_level)
    summary_pooled <- summary_pooled %>%
      dplyr::mutate(
```

```
          OR = exp(estimate),
          OR_low = exp(estimate - 1.96 * std.error),
          OR_high = exp(estimate + 1.96 * std.error)
        )
      matched_idx <- rep(FALSE, nrow(summary_pooled))
      if(length(mm_names) > 0) {
        matched_idx <- sapply(summary_pooled$term, function(t) {
          any(sapply(mm_names, function(m) {
            grepl(m, t, fixed = TRUE)
          }))
        })
      }
      if (!any(matched_idx)) {
        matched_idx <- grepl(var, summary_pooled$term, fixed = TRUE)
      }
      outcome_rows <- summary_pooled[matched_idx, , drop = FALSE]
      outcome_rows <- outcome_rows %>% mutate(Variable = var, model_call
= call_text)
      list(ok = TRUE, var = var, fit = fit, pooled = pooled,
           summary_pooled = summary_pooled, outcome_rows =
outcome_rows)
    }, error = function(e) {
      warning("Variable", var, " Failure： ", e$message)
      list(ok = FALSE, var = var, error = e$message, outcome_rows = NULL)
    })
    results_list[[var]] <- res_obj
}
all_outcomes <- do.call(rbind, lapply(results_list, function(x) {
    if(!is.null(x) && isTRUE(x$ok) && !is.null(x$outcome_rows) &&
nrow(x$outcome_rows) > 0) {
      df <- x$outcome_rows
      df %>% dplyr::select(Variable, term, estimate, std.error, statistic, df,
p.value, OR, OR_low, OR_high, model_call)
    } else {
      NULL
    }
}))
if (is.null(all_outcomes) || nrow(all_outcomes) == 0) {
    stop("Failed to match results")
}
all_outcomes <- all_outcomes %>%
    mutate(
      p_adj_BH = p.adjust(p.value, method = "BH"),
```

```
    p_adj_bonf = p.adjust(p.value, method = "bonferroni")
  )
all_outcomes <- all_outcomes %>%
  mutate(
    OR_fmt = ifelse(!is.na(OR), sprintf("%.3f", OR), NA_character_),
    CI_fmt = ifelse(!is.na(OR_low) & !is.na(OR_high),
                    paste0(sprintf("%.3f", OR_low), " - ", sprintf("%.3f",
OR_high)),
                    NA_character_),
    p_raw_fmt = ifelse(!is.na(p.value), sprintf("%.3f", p.value),
NA_character_),
    p_adj_BH_fmt = ifelse(!is.na(p_adj_BH), sprintf("%.3f", p_adj_BH),
NA_character_),
    p_adj_bonf_fmt = ifelse(!is.na(p_adj_bonf), sprintf("%.3f", p_adj_bonf),
NA_character_)
  ) %>%
  dplyr::select(Variable, term, OR_fmt, CI_fmt, p_raw_fmt, p_adj_BH_fmt,
p_adj_bonf_fmt, model_call)
# Saving results
write.csv(all_outcomes, "Univariate_ordinal_LOEU.csv", row.names = FALSE)


######### LOE-CN vs. LOE-CI Binomial Regression ############
# Defining Binomial Outcome
data_LOE_Binomial <- subset(data, Outcome != "HC")
data_LOE_Binomial$Outcome_LOE <-
factor(data_LOE_Binomial$Outcome_LOE, levels = c("LOE-CN", "LOE-CI"),
ordered = TRUE)
######### MICE Multiple Imputation
# Selecting the variables to be included in MICE dataset
Variables_overall <- c("Age", "Sex", "Edu_yrs", "Overweight", "BMI",
"Obesity", "Onset_year", "Active_Sz", "EpiDur", "BTCS", "Sleep.disorder",
"Mood.disorder", "HBP", "DM", "Lipidemia", "CHD", "Smoking", "Stroke",
"Trauma", "Surgery", "Cer_Infection", "Tumor", "Alcohol_Consume",
"Image_Pos", "Cerebral_Atrophy", "HS", "SVD", "Vascular_Mal", "Malacia",
"CurrMed", "EEG_Con", "SWA", "G.theta", "G.delta", "FS.left", "FS.right",
"FS.bilateral", "TIRDA", "TIRDA_total", "Ant_Tem_IED", "Oht_Tem_IED",
"Extra_Tem_IED")
formula <- as.formula(paste("Outcome_LOE ~", paste(Variables_overall,
collapse = " + ")))
data_LOE_Binomial <- data_LOE_Binomial[, c("Outcome_LOE",
Variables_overall)]
# Defining imputation methods
md.pattern(data_LOE_Binomial)
```

```
vars_with_missing <-
names(data_LOE_Binomial)[colSums(is.na(data_LOE_Binomial)) > 0]
vars_without_missing <- setdiff(names(data_LOE_Binomial),
vars_with_missing)
methods <- make.method(data_LOE_Binomial)
methods["BMI"] <- "pmm"
methods["Obesity"] <- "logreg"
methods["Edu_yrs"] <- "pmm"
methods["Active_Sz"] <- "logreg"
methods["Image_Pos"] <- "logreg"
methods["Cerebral_Atrophy"] <- "logreg"
methods["HS"] <- "logreg"
methods["SVD"] <- "logreg"
methods["Vascular_Mal"] <- "logreg"
methods["Malacia"] <- "logreg"
pred_matrix <- make.predictorMatrix(data_LOE_Binomial)
pred_matrix[, vars_without_missing] <- 0
# MICE
imp <- mice(data_LOE_Binomial, m =10, method = methods, seed = 1234,
predictorMatrix = pred_matrix)
summary(imp)
######### Univariate Binomial Logistic Regression and FDR correction
exploratory_vars <- c("Age", "Sex", "Edu_yrs", "Obesity", "Onset_year",
"Active_Sz", "EpiDur", "BTCS", "HBP", "DM", "Lipidemia", "CHD", "Smoking",
"Alcohol_Consume", "Sleep.disorder", "Mood.disorder", "Stroke", "Trauma",
"Surgery", "Cer_Infection", "Tumor", "Image_Pos", "Cerebral_Atrophy", "HS",
"SVD", "Vascular_Mal", "Malacia", "EEG_Con", "SWA", "G.theta", "G.delta",
"FS.left", "FS.right", "FS.bilateral", "TIRDA_total", "TIRDA", "Ant_Tem_IED",
"Oht_Tem_IED", "Extra_Tem_IED")
outcome_name <- "Outcome_LOE"
conf_level <- 0.95
results_list <- list()
for (var in exploratory_vars) {
  formula_text <- paste0(outcome_name, " ~ `", var, "`")
  mm_names <- character(0)
  try({
    mm <- model.matrix(as.formula(formula_text), data = comp1)
    mm_names <- colnames(mm)
    mm_names <- setdiff(mm_names, "(Intercept)")
  }, silent = TRUE)
  res_obj <- tryCatch({
    call_text <- paste0("with(imp, glm(", formula_text, ", family = binomial))")
    fit <- eval(parse(text = call_text))
    pooled <- pool(fit)
```

```
      summary_pooled <- summary(pooled, conf.int = TRUE, conf.level =
conf_level)
      summary_pooled <- summary_pooled %>%
         dplyr::mutate(
            OR = exp(estimate),
            OR_low = exp(estimate - 1.96 * std.error),
            OR_high = exp(estimate + 1.96 * std.error)
         )
      matched_idx <- rep(FALSE, nrow(summary_pooled))
      if(length(mm_names) > 0) {
         matched_idx <- sapply(summary_pooled$term, function(t) {
            any(sapply(mm_names, function(m) {
               grepl(m, t, fixed = TRUE)
            }))
         })
      }
      if (!any(matched_idx)) {
         matched_idx <- grepl(var, summary_pooled$term, fixed = TRUE)
      }
      outcome_rows <- summary_pooled[matched_idx, , drop = FALSE]
      outcome_rows <- outcome_rows %>% mutate(Variable = var, model_call
= call_text)
      list(ok = TRUE, var = var, fit = fit, pooled = pooled,
             summary_pooled = summary_pooled, outcome_rows =
outcome_rows)
   }, error = function(e) {
      warning("Variable", var, " Failure： ", e$message)
      list(ok = FALSE, var = var, error = e$message, outcome_rows = NULL)
   })
   results_list[[var]] <- res_obj
}
all_outcomes <- do.call(rbind, lapply(results_list, function(x) {
   if(!is.null(x) && isTRUE(x$ok) && !is.null(x$outcome_rows) &&
nrow(x$outcome_rows) > 0) {
      df <- x$outcome_rows
      df %>% dplyr::select(Variable, term, estimate, std.error, statistic, df,
p.value, OR, OR_low, OR_high, model_call)
   } else {
      NULL
   }
}))
if (is.null(all_outcomes) || nrow(all_outcomes) == 0) {
   stop("Failed to match results")
```

```
}
all_outcomes <- all_outcomes %>%
  mutate(
    p_adj_BH = p.adjust(p.value, method = "BH"),
    p_adj_bonf = p.adjust(p.value, method = "bonferroni")
  )
all_outcomes <- all_outcomes %>%
  mutate(
    OR_fmt = ifelse(!is.na(OR), sprintf("%.3f", OR), NA_character_),
    CI_fmt = ifelse(!is.na(OR_low) & !is.na(OR_high),
                    paste0(sprintf("%.3f", OR_low), " - ", sprintf("%.3f",
OR_high)),
                    NA_character_),
    p_raw_fmt = ifelse(!is.na(p.value), sprintf("%.3f", p.value),
NA_character_),
    p_adj_BH_fmt = ifelse(!is.na(p_adj_BH), sprintf("%.3f", p_adj_BH),
NA_character_),
    p_adj_bonf_fmt = ifelse(!is.na(p_adj_bonf), sprintf("%.3f", p_adj_bonf),
NA_character_)
  ) %>%
  dplyr::select(Variable, term, OR_fmt, CI_fmt, p_raw_fmt, p_adj_BH_fmt,
p_adj_bonf_fmt, model_call)
write.csv(all_outcomes, "Univariate_binomial_results_LOE_with_padj.csv",
row.names = FALSE)

######### Multivariate Binomial Logistic Regression and FDR correction,
Controlling for Demographics only
exploratory_vars <- c("Obesity", "Onset_year", "Active_Sz", "EpiDur", "BTCS",
"HBP", "DM", "Lipidemia", "CHD", "Smoking", "Alcohol_Consume",
"Sleep.disorder", "Mood.disorder", "Stroke", "Trauma", "Surgery",
"Cer_Infection", "Tumor", "Image_Pos", "Cerebral_Atrophy", "HS", "SVD",
"Vascular_Mal", "Malacia", "EEG_Con", "SWA", "G.theta", "G.delta", "FS.left",
"FS.right", "FS.bilateral", "TIRDA_total", "TIRDA", "Ant_Tem_IED",
"Oht_Tem_IED", "Extra_Tem_IED")
confounders <- c("Age", "Sex", "Edu_yrs")
outcome_name <- "Outcome_LOE"
conf_level <- 0.95
results_list <- list()
for (var in exploratory_vars) {
  formula_text <- paste0(outcome_name, " ~ `", var, "` + ",
paste(confounders, collapse = " + "))
  mm_names <- character(0)
  try({
    mm <- model.matrix(as.formula(formula_text), data = comp1)
```

```
    mm_names <- colnames(mm)
    mm_names <- setdiff(mm_names, "(Intercept)")
  }, silent = TRUE)
  res_obj <- tryCatch({
    call_text <- paste0("with(imp, glm(", formula_text, ", family = binomial))")
    fit <- eval(parse(text = call_text))
    pooled <- pool(fit)
    summary_pooled <- summary(pooled, conf.int = TRUE, conf.level =
conf_level)
    summary_pooled <- summary_pooled %>%
      dplyr::mutate(
        OR = exp(estimate),
        OR_low = exp(estimate - 1.96 * std.error),
        OR_high = exp(estimate + 1.96 * std.error)
      )
    matched_idx <- rep(FALSE, nrow(summary_pooled))
    matched_idx <- grepl(var, summary_pooled$term, fixed = TRUE)
    if (any(matched_idx)) {
      confounder_pattern <- paste(confounders, collapse = "|")
      matched_idx <- matched_idx & !grepl(confounder_pattern,
summary_pooled$term, fixed = FALSE)
    }
    outcome_rows <- summary_pooled[matched_idx, , drop = FALSE]
    outcome_rows <- outcome_rows %>% mutate(Variable = var, model_call
= call_text)
    list(ok = TRUE, var = var, fit = fit, pooled = pooled,
         summary_pooled = summary_pooled, outcome_rows =
outcome_rows)
  }, error = function(e) {
    warning("Variable", var, " Failure： ", e$message)
    list(ok = FALSE, var = var, error = e$message, outcome_rows = NULL)
  })
  results_list[[var]] <- res_obj
}
all_outcomes <- do.call(rbind, lapply(results_list, function(x) {
  if(!is.null(x) && isTRUE(x$ok) && !is.null(x$outcome_rows) &&
nrow(x$outcome_rows) > 0) {
    df <- x$outcome_rows
    df %>% dplyr::select(Variable, term, estimate, std.error, statistic, df,
p.value, OR, OR_low, OR_high, model_call)
  } else {
    NULL
  }
```

```
}))
if (is.null(all_outcomes) || nrow(all_outcomes) == 0) {
   stop("Failed to match results")
}
all_outcomes <- all_outcomes %>%
   mutate(
      p_adj_BH = p.adjust(p.value, method = "BH"),
      p_adj_bonf = p.adjust(p.value, method = "bonferroni")
   )
all_outcomes <- all_outcomes %>%
   mutate(
      OR_fmt = ifelse(!is.na(OR), sprintf("%.3f", OR), NA_character_),
      CI_fmt = ifelse(!is.na(OR_low) & !is.na(OR_high),
                        paste0(sprintf("%.3f", OR_low), " - ", sprintf("%.3f",
OR_high)),
                        NA_character_),
      p_raw_fmt = ifelse(!is.na(p.value), sprintf("%.3f", p.value),
NA_character_),
      p_adj_BH_fmt = ifelse(!is.na(p_adj_BH), sprintf("%.3f", p_adj_BH),
NA_character_),
      p_adj_bonf_fmt = ifelse(!is.na(p_adj_bonf), sprintf("%.3f", p_adj_bonf),
NA_character_)
   ) %>%
   dplyr::select(Variable, term, OR_fmt, CI_fmt, p_raw_fmt, p_adj_BH_fmt,
p_adj_bonf_fmt, model_call)
write.csv(all_outcomes,
"Multivariate_demographics_binomial_results_LOE_with_padj.csv",
row.names = FALSE)

######### Multivariate Binomial Logistic Regression and FDR correction,
Controlling for all Confounders
exploratory_vars <- c("EEG_Con", "SWA", "G.theta", "G.delta", "FS.left",
"FS.right", "FS.bilateral", "TIRDA_total", "TIRDA", "Ant_Tem_IED",
"Oht_Tem_IED", "Extra_Tem_IED")
confounders <- c("Age", "Sex", "Edu_yrs", "Obesity", "EpiDur", "Active_Sz",
"BTCS", "HBP", "DM", "Lipidemia", "Smoking", "Alcohol_Consume",
"CurrMed",    "Mood.disorder", "Cerebral_Atrophy", "SVD")
outcome_name <- "Outcome_LOE"
conf_level <- 0.95
results_list <- list()
for (var in exploratory_vars) {
   formula_text <- paste0(outcome_name, " ~ `", var, "` + ",
paste(confounders, collapse = " + "))
   mm_names <- character(0)
```

```
   try({
      mm <- model.matrix(as.formula(formula_text), data = comp1)
      mm_names <- colnames(mm)
      mm_names <- setdiff(mm_names, "(Intercept)")
   }, silent = TRUE)
   res_obj <- tryCatch({
      call_text <- paste0("with(imp, glm(", formula_text, ", family = binomial))")
      fit <- eval(parse(text = call_text))
      pooled <- pool(fit)
      summary_pooled <- summary(pooled, conf.int = TRUE, conf.level =
conf_level)
      summary_pooled <- summary_pooled %>%
         dplyr::mutate(
            OR = exp(estimate),
            OR_low = exp(estimate - 1.96 * std.error),
            OR_high = exp(estimate + 1.96 * std.error)
         )
      matched_idx <- rep(FALSE, nrow(summary_pooled))
      matched_idx <- grepl(var, summary_pooled$term, fixed = TRUE)
      if (any(matched_idx)) {
         confounder_pattern <- paste(confounders, collapse = "|")
         matched_idx <- matched_idx & !grepl(confounder_pattern,
summary_pooled$term, fixed = FALSE)
      }
      outcome_rows <- summary_pooled[matched_idx, , drop = FALSE]
      outcome_rows <- outcome_rows %>% mutate(Variable = var, model_call
= call_text)
      list(ok = TRUE, var = var, fit = fit, pooled = pooled,
             summary_pooled = summary_pooled, outcome_rows =
outcome_rows)
   }, error = function(e) {
      warning("Variable", var, " Failure： ", e$message)
      list(ok = FALSE, var = var, error = e$message, outcome_rows = NULL)
   })
   results_list[[var]] <- res_obj
}
all_outcomes <- do.call(rbind, lapply(results_list, function(x) {
   if(!is.null(x) && isTRUE(x$ok) && !is.null(x$outcome_rows) &&
nrow(x$outcome_rows) > 0) {
      df <- x$outcome_rows
      df %>% dplyr::select(Variable, term, estimate, std.error, statistic, df,
p.value, OR, OR_low, OR_high, model_call)
   } else {
```

```
      NULL
    }
  }))
  if (is.null(all_outcomes) || nrow(all_outcomes) == 0) {
    stop("Failed to match results")
  }
  all_outcomes <- all_outcomes %>%
    mutate(
      p_adj_BH = p.adjust(p.value, method = "BH"),
      p_adj_bonf = p.adjust(p.value, method = "bonferroni")
    )
  all_outcomes <- all_outcomes %>%
    mutate(
      OR_fmt = ifelse(!is.na(OR), sprintf("%.3f", OR), NA_character_),
      CI_fmt = ifelse(!is.na(OR_low) & !is.na(OR_high),
                      paste0(sprintf("%.3f", OR_low), " - ", sprintf("%.3f",
OR_high)),
                      NA_character_),
      p_raw_fmt = ifelse(!is.na(p.value), sprintf("%.3f", p.value),
NA_character_),
      p_adj_BH_fmt = ifelse(!is.na(p_adj_BH), sprintf("%.3f", p_adj_BH),
NA_character_),
      p_adj_bonf_fmt = ifelse(!is.na(p_adj_bonf), sprintf("%.3f", p_adj_bonf),
NA_character_)
    ) %>%
    dplyr::select(Variable, term, OR_fmt, CI_fmt, p_raw_fmt, p_adj_BH_fmt,
p_adj_bonf_fmt, model_call)
  write.csv(all_outcomes,
"Multivariate_confounders_binomial_results_LOE_with_padj.csv", row.names
= FALSE)


######### LOEU-CN vs. LOEU-CI Binomial Regression ############
# Defining Binomial Outcome
data_LOEU_Binomial <- subset(data, Outcome != "HC")
data_LOEU_Binomial <- subset(data_LOEU_Binomial, Outcome !=
"nonLOEU-CN")
data_LOEU_Binomial <- subset(data_LOEU_Binomial, Outcome !=
"nonLOEU-CI")
data_LOEU_Binomial$Outcome <- factor(data_LOEU_Binomial$Outcome,
levels = c("LOEU-CN", "LOEU-CI"), ordered = TRUE)
######### MICE Multiple Imputation
# Selecting the variables to be included in MICE dataset
Variables_overall <- c("Age", "Sex", "Edu_yrs", "Overweight", "BMI",
```

```
"Obesity", "Onset_year", "Active_Sz", "EpiDur", "BTCS", "Sleep.disorder",
"Mood.disorder", "HBP", "DM", "Lipidemia", "CHD", "Smoking", "Stroke",
"Trauma", "Surgery", "Cer_Infection", "Tumor", "Alcohol_Consume",
"Image_Pos", "Cerebral_Atrophy", "HS", "SVD", "Vascular_Mal", "Malacia",
"CurrMed", "EEG_Con", "SWA", "G.theta", "G.delta", "FS.left", "FS.right",
"FS.bilateral", "TIRDA", "TIRDA_total", "Ant_Tem_IED", "Oht_Tem_IED",
"Extra_Tem_IED")
formula <- as.formula(paste("Outcome ~", paste(Variables_overall, collapse =
" + ")))
data_LOEU_Binomial <- data_LOEU_Binomial[, c("Outcome",
Variables_overall)]
# Defining imputation methods
md.pattern(data_LOEU_Binomial)
vars_with_missing <-
names(data_LOEU_Binomial)[colSums(is.na(data_LOEU_Binomial)) > 0]
vars_without_missing <- setdiff(names(data_LOEU_Binomial),
vars_with_missing)
methods <- make.method(data_LOEU_Binomial)
methods["BMI"] <- "pmm"
methods["Obesity"] <- "logreg"
methods["Edu_yrs"] <- "pmm"
methods["Active_Sz"] <- "logreg"
methods["Image_Pos"] <- "logreg"
methods["Cerebral_Atrophy"] <- "logreg"
methods["HS"] <- "logreg"
methods["SVD"] <- "logreg"
methods["Vascular_Mal"] <- "logreg"
methods["Malacia"] <- "logreg"
pred_matrix <- make.predictorMatrix(data_LOEU_Binomial)
pred_matrix[, vars_without_missing] <- 0
# MICE
imp <- mice(data_LOEU_Binomial, m =10, method = methods, seed = 1234,
predictorMatrix = pred_matrix)
summary(imp)
######### Univariate Binomial Logistic Regression and FDR correction
exploratory_vars <- c("Age", "Sex", "Edu_yrs", "Obesity", "Onset_year",
"Active_Sz", "EpiDur", "BTCS", "HBP", "DM", "Lipidemia", "CHD", "Smoking",
"Alcohol_Consume", "Sleep.disorder", "Mood.disorder", "Image_Pos",
"Cerebral_Atrophy", "HS", "SVD", "Vascular_Mal", "EEG_Con", "SWA",
"G.theta", "G.delta", "FS.left", "FS.right", "FS.bilateral", "TIRDA_total",
"TIRDA", "Ant_Tem_IED", "Oht_Tem_IED", "Extra_Tem_IED")
outcome_name <- "Outcome"
conf_level <- 0.95
results_list <- list()
```

```
for (var in exploratory_vars) {
  formula_text <- paste0(outcome_name, " ~ `", var, "`")
  mm_names <- character(0)
  try({
    mm <- model.matrix(as.formula(formula_text), data = comp1)
    mm_names <- colnames(mm)
    mm_names <- setdiff(mm_names, "(Intercept)")
  }, silent = TRUE)
  res_obj <- tryCatch({
    call_text <- paste0("with(imp, glm(", formula_text, ", family = binomial))")
    fit <- eval(parse(text = call_text))
    pooled <- pool(fit)
    summary_pooled <- summary(pooled, conf.int = TRUE, conf.level =
conf_level)
    summary_pooled <- summary_pooled %>%
      dplyr::mutate(
        OR = exp(estimate),
        OR_low = exp(estimate - 1.96 * std.error),
        OR_high = exp(estimate + 1.96 * std.error)
      )
    matched_idx <- rep(FALSE, nrow(summary_pooled))
    if(length(mm_names) > 0) {
      matched_idx <- sapply(summary_pooled$term, function(t) {
        any(sapply(mm_names, function(m) {
          grepl(m, t, fixed = TRUE)
        }))
      })
    }
    if (!any(matched_idx)) {
      matched_idx <- grepl(var, summary_pooled$term, fixed = TRUE)
    }
    outcome_rows <- summary_pooled[matched_idx, , drop = FALSE]
    outcome_rows <- outcome_rows %>% mutate(Variable = var, model_call
= call_text)
    list(ok = TRUE, var = var, fit = fit, pooled = pooled,
         summary_pooled = summary_pooled, outcome_rows =
outcome_rows)
  }, error = function(e) {
    warning("Variable", var, " Failure： ", e$message)
    list(ok = FALSE, var = var, error = e$message, outcome_rows = NULL)
  })
  results_list[[var]] <- res_obj
}
```

```
all_outcomes <- do.call(rbind, lapply(results_list, function(x) {
  if(!is.null(x) && isTRUE(x$ok) && !is.null(x$outcome_rows) &&
nrow(x$outcome_rows) > 0) {
    df <- x$outcome_rows
    df %>% dplyr::select(Variable, term, estimate, std.error, statistic, df,
p.value, OR, OR_low, OR_high, model_call)
  } else {
    NULL
  }
}))
if (is.null(all_outcomes) || nrow(all_outcomes) == 0) {
  stop("Failed to match results")
}
all_outcomes <- all_outcomes %>%
  mutate(
    p_adj_BH = p.adjust(p.value, method = "BH"),
    p_adj_bonf = p.adjust(p.value, method = "bonferroni")
  )
all_outcomes <- all_outcomes %>%
  mutate(
    OR_fmt = ifelse(!is.na(OR), sprintf("%.3f", OR), NA_character_),
    CI_fmt = ifelse(!is.na(OR_low) & !is.na(OR_high),
                    paste0(sprintf("%.3f", OR_low), " - ", sprintf("%.3f",
OR_high)),
                    NA_character_),
    p_raw_fmt = ifelse(!is.na(p.value), sprintf("%.3f", p.value),
NA_character_),
    p_adj_BH_fmt = ifelse(!is.na(p_adj_BH), sprintf("%.3f", p_adj_BH),
NA_character_),
    p_adj_bonf_fmt = ifelse(!is.na(p_adj_bonf), sprintf("%.3f", p_adj_bonf),
NA_character_)
  ) %>%
  dplyr::select(Variable, term, OR_fmt, CI_fmt, p_raw_fmt, p_adj_BH_fmt,
p_adj_bonf_fmt, model_call)
write.csv(all_outcomes, "Univariate_binomial_results_LOEU_with_padj.csv",
row.names = FALSE)

######### Multivariate Binomial Logistic Regression and FDR correction,
Controlling for Demographics only
exploratory_vars <- c("Obesity", "Onset_year", "Active_Sz", "EpiDur", "BTCS",
"HBP", "DM", "Lipidemia", "CHD", "Smoking", "Alcohol_Consume",
"Sleep.disorder", "Mood.disorder", "Image_Pos", "Cerebral_Atrophy", "HS",
"SVD", "Vascular_Mal", "EEG_Con", "SWA", "G.theta", "G.delta", "FS.left",
"FS.right", "FS.bilateral", "TIRDA_total", "TIRDA", "Ant_Tem_IED",
```

```
"Oht_Tem_IED", "Extra_Tem_IED")
confounders <- c("Age", "Sex", "Edu_yrs")
outcome_name <- "Outcome"
conf_level <- 0.95
results_list <- list()
for (var in exploratory_vars) {
  formula_text <- paste0(outcome_name, " ~ `", var, "` + ",
paste(confounders, collapse = " + "))
  mm_names <- character(0)
  try({
    mm <- model.matrix(as.formula(formula_text), data = comp1)
    mm_names <- colnames(mm)
    mm_names <- setdiff(mm_names, "(Intercept)")
  }, silent = TRUE)
  res_obj <- tryCatch({
    call_text <- paste0("with(imp, glm(", formula_text, ", family = binomial))")
    fit <- eval(parse(text = call_text))
    pooled <- pool(fit)
    summary_pooled <- summary(pooled, conf.int = TRUE, conf.level =
conf_level)
    summary_pooled <- summary_pooled %>%
      dplyr::mutate(
        OR = exp(estimate),
        OR_low = exp(estimate - 1.96 * std.error),
        OR_high = exp(estimate + 1.96 * std.error)
      )
    matched_idx <- rep(FALSE, nrow(summary_pooled))
    matched_idx <- grepl(var, summary_pooled$term, fixed = TRUE)
    if (any(matched_idx)) {
      confounder_pattern <- paste(confounders, collapse = "|")
      matched_idx <- matched_idx & !grepl(confounder_pattern,
summary_pooled$term, fixed = FALSE)
    }
    outcome_rows <- summary_pooled[matched_idx, , drop = FALSE]
    outcome_rows <- outcome_rows %>% mutate(Variable = var, model_call
= call_text)
    list(ok = TRUE, var = var, fit = fit, pooled = pooled,
         summary_pooled = summary_pooled, outcome_rows =
outcome_rows)
  }, error = function(e) {

    warning("Variable", var, " Failure： ", e$message)

    list(ok = FALSE, var = var, error = e$message, outcome_rows = NULL)
  })
```

```
    results_list[[var]] <- res_obj
}
all_outcomes <- do.call(rbind, lapply(results_list, function(x) {
    if(!is.null(x) && isTRUE(x$ok) && !is.null(x$outcome_rows) &&
nrow(x$outcome_rows) > 0) {
        df <- x$outcome_rows
        df %>% dplyr::select(Variable, term, estimate, std.error, statistic, df,
p.value, OR, OR_low, OR_high, model_call)
    } else {
        NULL
    }
}))
if (is.null(all_outcomes) || nrow(all_outcomes) == 0) {
    stop("Failed to match results")
}
all_outcomes <- all_outcomes %>%
    mutate(
        p_adj_BH = p.adjust(p.value, method = "BH"),
        p_adj_bonf = p.adjust(p.value, method = "bonferroni")
    )
all_outcomes <- all_outcomes %>%
    mutate(
        OR_fmt = ifelse(!is.na(OR), sprintf("%.3f", OR), NA_character_),
        CI_fmt = ifelse(!is.na(OR_low) & !is.na(OR_high),
                                paste0(sprintf("%.3f", OR_low), " - ", sprintf("%.3f",
OR_high)),
                                NA_character_),
        p_raw_fmt = ifelse(!is.na(p.value), sprintf("%.3f", p.value),
NA_character_),
        p_adj_BH_fmt = ifelse(!is.na(p_adj_BH), sprintf("%.3f", p_adj_BH),
NA_character_),
        p_adj_bonf_fmt = ifelse(!is.na(p_adj_bonf), sprintf("%.3f", p_adj_bonf),
NA_character_)
    ) %>%
    dplyr::select(Variable, term, OR_fmt, CI_fmt, p_raw_fmt, p_adj_BH_fmt,
p_adj_bonf_fmt, model_call)
write.csv(all_outcomes,
"Multivariate_demographics_binomial_results_LOEU_with_padj.csv",
row.names = FALSE)

######### Multivariate Binomial Logistic Regression and FDR correction,
Controlling for all Confounders
exploratory_vars <- c("EEG_Con", "SWA", "G.theta", "G.delta", "FS.left",
"FS.right", "FS.bilateral", "TIRDA_total", "TIRDA", "Ant_Tem_IED",
```

```
"Oht_Tem_IED", "Extra_Tem_IED")
confounders <- c("Age", "Sex", "Edu_yrs", "Obesity", "EpiDur", "Active_Sz",
"BTCS", "HBP", "DM", "Lipidemia", "Smoking", "Alcohol_Consume",
"CurrMed",    "Mood.disorder", "Cerebral_Atrophy", "SVD")
outcome_name <- "Outcome"
conf_level <- 0.95
results_list <- list()
for (var in exploratory_vars) {
  formula_text <- paste0(outcome_name, " ~ `", var, "` + ",
paste(confounders, collapse = " + "))
  mm_names <- character(0)
  try({
    mm <- model.matrix(as.formula(formula_text), data = comp1)
    mm_names <- colnames(mm)
    mm_names <- setdiff(mm_names, "(Intercept)")
  }, silent = TRUE)
  res_obj <- tryCatch({
    call_text <- paste0("with(imp, glm(", formula_text, ", family = binomial))")
    fit <- eval(parse(text = call_text))
    pooled <- pool(fit)
    summary_pooled <- summary(pooled, conf.int = TRUE, conf.level =
conf_level)
    summary_pooled <- summary_pooled %>%
      dplyr::mutate(
        OR = exp(estimate),
        OR_low = exp(estimate - 1.96 * std.error),
        OR_high = exp(estimate + 1.96 * std.error)
      )
    matched_idx <- rep(FALSE, nrow(summary_pooled))
    matched_idx <- grepl(var, summary_pooled$term, fixed = TRUE)
    if (any(matched_idx)) {
      confounder_pattern <- paste(confounders, collapse = "|")
      matched_idx <- matched_idx & !grepl(confounder_pattern,
summary_pooled$term, fixed = FALSE)
    }
    outcome_rows <- summary_pooled[matched_idx, , drop = FALSE]
    outcome_rows <- outcome_rows %>% mutate(Variable = var, model_call
= call_text)
    list(ok = TRUE, var = var, fit = fit, pooled = pooled,
         summary_pooled = summary_pooled, outcome_rows =
outcome_rows)
  }, error = function(e) {
    warning("Variable", var, " Failure：", e$message)
```

```
        list(ok = FALSE, var = var, error = e$message, outcome_rows = NULL)
    })
    results_list[[var]] <- res_obj
}
all_outcomes <- do.call(rbind, lapply(results_list, function(x) {
    if(!is.null(x) && isTRUE(x$ok) && !is.null(x$outcome_rows) &&
nrow(x$outcome_rows) > 0) {
        df <- x$outcome_rows
        df %>% dplyr::select(Variable, term, estimate, std.error, statistic, df,
p.value, OR, OR_low, OR_high, model_call)
    } else {
        NULL
    }
}))
if (is.null(all_outcomes) || nrow(all_outcomes) == 0) {
    stop("Failed to match results")
}
all_outcomes <- all_outcomes %>%
    mutate(
        p_adj_BH = p.adjust(p.value, method = "BH"),
        p_adj_bonf = p.adjust(p.value, method = "bonferroni")
    )
all_outcomes <- all_outcomes %>%
    mutate(
        OR_fmt = ifelse(!is.na(OR), sprintf("%.3f", OR), NA_character_),
        CI_fmt = ifelse(!is.na(OR_low) & !is.na(OR_high),
                        paste0(sprintf("%.3f", OR_low), " - ", sprintf("%.3f",
OR_high)),
                        NA_character_),
        p_raw_fmt = ifelse(!is.na(p.value), sprintf("%.3f", p.value),
NA_character_),
        p_adj_BH_fmt = ifelse(!is.na(p_adj_BH), sprintf("%.3f", p_adj_BH),
NA_character_),
        p_adj_bonf_fmt = ifelse(!is.na(p_adj_bonf), sprintf("%.3f", p_adj_bonf),
NA_character_)
    ) %>%
    dplyr::select(Variable, term, OR_fmt, CI_fmt, p_raw_fmt, p_adj_BH_fmt,
p_adj_bonf_fmt, model_call)
write.csv(all_outcomes,
"Multivariate_confounders_binomial_results_LOEU_with_padj.csv",
row.names = FALSE)
```

```
######### LOED-CN vs. LOED-CI Binomial Regression ###########
# Defining Binomial Outcome
data_LOED_Binomial <- subset(data, Outcome != "HC")
data_LOED_Binomial <- subset(data_LOED_Binomial, Outcome != "LOEU-
CN")
data_LOED_Binomial <- subset(data_LOED_Binomial, Outcome != "LOEU-
CI")
data_LOED_Binomial$Outcome <- factor(data_LOED_Binomial$Outcome,
levels = c("nonLOEU-CN", "nonLOEU-CI"), ordered = TRUE)
######### MICE Multiple Imputation
# Selecting the variables to be included in MICE dataset
Variables_overall <- c("Age", "Sex", "Edu_yrs", "Overweight", "BMI",
"Obesity", "Onset_year", "Active_Sz", "EpiDur", "BTCS", "Sleep.disorder",
"Mood.disorder", "HBP", "DM", "Lipidemia", "CHD", "Smoking", "Stroke",
"Trauma", "Surgery", "Cer_Infection", "Tumor", "Alcohol_Consume",
"Image_Pos", "Cerebral_Atrophy", "HS", "SVD", "Vascular_Mal", "Malacia",
"CurrMed", "EEG_Con", "SWA", "G.theta", "G.delta", "FS.left", "FS.right",
"FS.bilateral", "TIRDA", "TIRDA_total", "Ant_Tem_IED", "Oht_Tem_IED",
"Extra_Tem_IED")
formula <- as.formula(paste("Outcome ~", paste(Variables_overall, collapse =
" + ")))
data_LOED_Binomial <- data_LOED_Binomial[, c("Outcome",
Variables_overall)]
# Defining imputation methods
md.pattern(data_LOED_Binomial)
vars_with_missing <-
names(data_LOED_Binomial)[colSums(is.na(data_LOED_Binomial)) > 0]
vars_without_missing <- setdiff(names(data_LOED_Binomial),
vars_with_missing)
methods <- make.method(data_LOED_Binomial)
methods["BMI"] <- "pmm"
methods["Obesity"] <- "logreg"
methods["Edu_yrs"] <- "pmm"
methods["Active_Sz"] <- "logreg"
methods["Image_Pos"] <- "logreg"
methods["Cerebral_Atrophy"] <- "logreg"
methods["HS"] <- "logreg"
methods["SVD"] <- "logreg"
methods["Vascular_Mal"] <- "logreg"
methods["Malacia"] <- "logreg"
pred_matrix <- make.predictorMatrix(data_LOED_Binomial)
pred_matrix[, vars_without_missing] <- 0
# MICE
imp <- mice(data_LOED_Binomial, m =10, method = methods, seed = 1234,
```

```
predictorMatrix = pred_matrix)
summary(imp)
######### Univariate Binomial Logistic Regression and FDR correction
exploratory_vars <- c("Age", "Sex", "Edu_yrs", "Obesity", "Onset_year",
"Active_Sz", "EpiDur", "BTCS", "HBP", "DM", "Lipidemia", "CHD", "Smoking",
"Alcohol_Consume", "Sleep.disorder", "Mood.disorder", "Stroke", "Trauma",
"Surgery", "Cer_Infection", "Tumor", "Image_Pos", "Cerebral_Atrophy", "HS",
"SVD", "Vascular_Mal", "Malacia", "EEG_Con", "SWA", "G.theta", "G.delta",
"FS.left", "FS.right", "FS.bilateral", "TIRDA_total", "TIRDA", "Ant_Tem_IED",
"Oht_Tem_IED", "Extra_Tem_IED")
outcome_name <- "Outcome"
conf_level <- 0.95
results_list <- list()
for (var in exploratory_vars) {
  formula_text <- paste0(outcome_name, " ~ `", var, "`")
  mm_names <- character(0)
  try({
    mm <- model.matrix(as.formula(formula_text), data = comp1)
    mm_names <- colnames(mm)
    mm_names <- setdiff(mm_names, "(Intercept)")
  }, silent = TRUE)
  res_obj <- tryCatch({
    call_text <- paste0("with(imp, glm(", formula_text, ", family = binomial))")
    fit <- eval(parse(text = call_text))
    pooled <- pool(fit)
    summary_pooled <- summary(pooled, conf.int = TRUE, conf.level =
conf_level)
    summary_pooled <- summary_pooled %>%
      dplyr::mutate(
        OR = exp(estimate),
        OR_low = exp(estimate - 1.96 * std.error),
        OR_high = exp(estimate + 1.96 * std.error)
      )
    matched_idx <- rep(FALSE, nrow(summary_pooled))
    if(length(mm_names) > 0) {
      matched_idx <- sapply(summary_pooled$term, function(t) {
        any(sapply(mm_names, function(m) {
          grepl(m, t, fixed = TRUE)
        }))
      })
    }
    if (!any(matched_idx)) {
      matched_idx <- grepl(var, summary_pooled$term, fixed = TRUE)
    }
```

```
    outcome_rows <- summary_pooled[matched_idx, , drop = FALSE]
    outcome_rows <- outcome_rows %>% mutate(Variable = var, model_call
= call_text)
    list(ok = TRUE, var = var, fit = fit, pooled = pooled,
         summary_pooled = summary_pooled, outcome_rows =
outcome_rows)
  }, error = function(e) {
    warning("Variable", var, " Failure： ", e$message)
    list(ok = FALSE, var = var, error = e$message, outcome_rows = NULL)
  })
  results_list[[var]] <- res_obj
}
all_outcomes <- do.call(rbind, lapply(results_list, function(x) {
  if(!is.null(x) && isTRUE(x$ok) && !is.null(x$outcome_rows) &&
nrow(x$outcome_rows) > 0) {
    df <- x$outcome_rows
    df %>% dplyr::select(Variable, term, estimate, std.error, statistic, df,
p.value, OR, OR_low, OR_high, model_call)
  } else {
    NULL
  }
}))
if (is.null(all_outcomes) || nrow(all_outcomes) == 0) {
  stop("Failed to match results")
}
all_outcomes <- all_outcomes %>%
  mutate(
    p_adj_BH = p.adjust(p.value, method = "BH"),
    p_adj_bonf = p.adjust(p.value, method = "bonferroni")
  )
all_outcomes <- all_outcomes %>%
  mutate(
    OR_fmt = ifelse(!is.na(OR), sprintf("%.3f", OR), NA_character_),
    CI_fmt = ifelse(!is.na(OR_low) & !is.na(OR_high),
                    paste0(sprintf("%.3f", OR_low), " - ", sprintf("%.3f",
OR_high)),
                    NA_character_),
    p_raw_fmt = ifelse(!is.na(p.value), sprintf("%.3f", p.value),
NA_character_),
    p_adj_BH_fmt = ifelse(!is.na(p_adj_BH), sprintf("%.3f", p_adj_BH),
NA_character_),
    p_adj_bonf_fmt = ifelse(!is.na(p_adj_bonf), sprintf("%.3f", p_adj_bonf),
NA_character_)
```

```
  ) %>%
  dplyr::select(Variable, term, OR_fmt, CI_fmt, p_raw_fmt, p_adj_BH_fmt,
p_adj_bonf_fmt, model_call)
write.csv(all_outcomes, "Univariate_binomial_results_LOED_with_padj.csv",
row.names = FALSE)

######### Multivariate Binomial Logistic Regression and FDR correction,
Controlling for Demographics only
exploratory_vars <- c("Obesity", "Onset_year", "Active_Sz", "EpiDur", "BTCS",
"HBP", "DM", "Lipidemia", "CHD", "Smoking", "Alcohol_Consume",
"Sleep.disorder", "Mood.disorder", "Stroke", "Trauma", "Surgery",
"Cer_Infection", "Tumor", "Image_Pos", "Cerebral_Atrophy", "HS", "SVD",
"Vascular_Mal", "Malacia", "EEG_Con", "SWA", "G.theta", "G.delta", "FS.left",
"FS.right", "FS.bilateral", "TIRDA_total", "TIRDA", "Ant_Tem_IED",
"Oht_Tem_IED", "Extra_Tem_IED")
confounders <- c("Age", "Sex", "Edu_yrs")
outcome_name <- "Outcome"
conf_level <- 0.95
results_list <- list()
for (var in exploratory_vars) {
  formula_text <- paste0(outcome_name, " ~ `", var, "` + ",
paste(confounders, collapse = " + "))
  mm_names <- character(0)
  try({
    mm <- model.matrix(as.formula(formula_text), data = comp1)
    mm_names <- colnames(mm)
    mm_names <- setdiff(mm_names, "(Intercept)")
  }, silent = TRUE)
  res_obj <- tryCatch({
    call_text <- paste0("with(imp, glm(", formula_text, ", family = binomial))")
    fit <- eval(parse(text = call_text))
    pooled <- pool(fit)
    summary_pooled <- summary(pooled, conf.int = TRUE, conf.level =
conf_level)
    summary_pooled <- summary_pooled %>%
      dplyr::mutate(
        OR = exp(estimate),
        OR_low = exp(estimate - 1.96 * std.error),
        OR_high = exp(estimate + 1.96 * std.error)
      )
    matched_idx <- rep(FALSE, nrow(summary_pooled))
    matched_idx <- grepl(var, summary_pooled$term, fixed = TRUE)
    if (any(matched_idx)) {
      confounder_pattern <- paste(confounders, collapse = "|")
```

```
        matched_idx <- matched_idx & !grepl(confounder_pattern,
summary_pooled$term, fixed = FALSE)
      }
      outcome_rows <- summary_pooled[matched_idx, , drop = FALSE]
      outcome_rows <- outcome_rows %>% mutate(Variable = var, model_call
= call_text)
      list(ok = TRUE, var = var, fit = fit, pooled = pooled,
           summary_pooled = summary_pooled, outcome_rows =
outcome_rows)
   }, error = function(e) {
      warning("Variable", var, " Failure： ", e$message)
      list(ok = FALSE, var = var, error = e$message, outcome_rows = NULL)
   })
   results_list[[var]] <- res_obj
}
all_outcomes <- do.call(rbind, lapply(results_list, function(x) {
   if(!is.null(x) && isTRUE(x$ok) && !is.null(x$outcome_rows) &&
nrow(x$outcome_rows) > 0) {
      df <- x$outcome_rows
      df %>% dplyr::select(Variable, term, estimate, std.error, statistic, df,
p.value, OR, OR_low, OR_high, model_call)
   } else {
      NULL
   }
}))
if (is.null(all_outcomes) || nrow(all_outcomes) == 0) {
   stop("Failed to match results")
}
all_outcomes <- all_outcomes %>%
   mutate(
      p_adj_BH = p.adjust(p.value, method = "BH"),
      p_adj_bonf = p.adjust(p.value, method = "bonferroni")
   )
all_outcomes <- all_outcomes %>%
   mutate(
      OR_fmt = ifelse(!is.na(OR), sprintf("%.3f", OR), NA_character_),
      CI_fmt = ifelse(!is.na(OR_low) & !is.na(OR_high),
                      paste0(sprintf("%.3f", OR_low), " - ", sprintf("%.3f",
OR_high)),
                      NA_character_),
      p_raw_fmt = ifelse(!is.na(p.value), sprintf("%.3f", p.value),
NA_character_),
      p_adj_BH_fmt = ifelse(!is.na(p_adj_BH), sprintf("%.3f", p_adj_BH),
```

```
NA_character_),
      p_adj_bonf_fmt = ifelse(!is.na(p_adj_bonf), sprintf("%.3f", p_adj_bonf),
NA_character_)
   ) %>%
   dplyr::select(Variable, term, OR_fmt, CI_fmt, p_raw_fmt, p_adj_BH_fmt,
p_adj_bonf_fmt, model_call)
write.csv(all_outcomes,
"Multivariate_demographics_binomial_results_LOED_with_padj.csv",
row.names = FALSE)

######### Multivariate Binomial Logistic Regression and FDR correction,
Controlling for all Confounders
exploratory_vars <- c("EEG_Con", "SWA", "G.theta", "G.delta", "FS.left",
"FS.right", "FS.bilateral", "TIRDA_total", "TIRDA", "Ant_Tem_IED",
"Oht_Tem_IED", "Extra_Tem_IED")
confounders <- c("Age", "Sex", "Edu_yrs", "Obesity", "EpiDur", "Active_Sz",
"BTCS", "HBP", "DM", "Lipidemia", "Smoking", "Alcohol_Consume",
"CurrMed",   "Mood.disorder", "Cerebral_Atrophy", "SVD")
outcome_name <- "Outcome"
conf_level <- 0.95
results_list <- list()
for (var in exploratory_vars) {
   formula_text <- paste0(outcome_name, " ~ `", var, "` + ",
paste(confounders, collapse = " + "))
   mm_names <- character(0)
   try({
      mm <- model.matrix(as.formula(formula_text), data = comp1)
      mm_names <- colnames(mm)
      mm_names <- setdiff(mm_names, "(Intercept)")
   }, silent = TRUE)
   res_obj <- tryCatch({
      call_text <- paste0("with(imp, glm(", formula_text, ", family = binomial))")
      fit <- eval(parse(text = call_text))
      pooled <- pool(fit)
      summary_pooled <- summary(pooled, conf.int = TRUE, conf.level =
conf_level)
      summary_pooled <- summary_pooled %>%
         dplyr::mutate(
            OR = exp(estimate),
            OR_low = exp(estimate - 1.96 * std.error),
            OR_high = exp(estimate + 1.96 * std.error)
         )
      matched_idx <- rep(FALSE, nrow(summary_pooled))
      matched_idx <- grepl(var, summary_pooled$term, fixed = TRUE)
```

```
      if (any(matched_idx)) {
         confounder_pattern <- paste(confounders, collapse = "|")
         matched_idx <- matched_idx & !grepl(confounder_pattern,
summary_pooled$term, fixed = FALSE)
      }
      outcome_rows <- summary_pooled[matched_idx, , drop = FALSE]
      outcome_rows <- outcome_rows %>% mutate(Variable = var, model_call
= call_text)
      list(ok = TRUE, var = var, fit = fit, pooled = pooled,
             summary_pooled = summary_pooled, outcome_rows =
outcome_rows)
   }, error = function(e) {
      warning("Variable", var, " Failure： ", e$message)
      list(ok = FALSE, var = var, error = e$message, outcome_rows = NULL)
   })
   results_list[[var]] <- res_obj
}
all_outcomes <- do.call(rbind, lapply(results_list, function(x) {
   if(!is.null(x) && isTRUE(x$ok) && !is.null(x$outcome_rows) &&
nrow(x$outcome_rows) > 0) {
      df <- x$outcome_rows
      df %>% dplyr::select(Variable, term, estimate, std.error, statistic, df,
p.value, OR, OR_low, OR_high, model_call)
   } else {
      NULL
   }
}))
if (is.null(all_outcomes) || nrow(all_outcomes) == 0) {
   stop("Failed to match results")
}
all_outcomes <- all_outcomes %>%
   mutate(
      p_adj_BH = p.adjust(p.value, method = "BH"),
      p_adj_bonf = p.adjust(p.value, method = "bonferroni")
   )
all_outcomes <- all_outcomes %>%
   mutate(
      OR_fmt = ifelse(!is.na(OR), sprintf("%.3f", OR), NA_character_),
      CI_fmt = ifelse(!is.na(OR_low) & !is.na(OR_high),
                      paste0(sprintf("%.3f", OR_low), " - ", sprintf("%.3f",
OR_high)),
                      NA_character_),
      p_raw_fmt = ifelse(!is.na(p.value), sprintf("%.3f", p.value),
```

```
NA_character_),
      p_adj_BH_fmt = ifelse(!is.na(p_adj_BH), sprintf("%.3f", p_adj_BH),
NA_character_),
      p_adj_bonf_fmt = ifelse(!is.na(p_adj_bonf), sprintf("%.3f", p_adj_bonf),
NA_character_)
   ) %>%
   dplyr::select(Variable, term, OR_fmt, CI_fmt, p_raw_fmt, p_adj_BH_fmt,
p_adj_bonf_fmt, model_call)
write.csv(all_outcomes,
"Multivariate_confounders_binomial_results_LOED_with_padj.csv",
row.names = FALSE)



######### LASSO regression for LOEU    ############
data_LOEU_Binomial <- subset(data, Outcome != "HC")
data_LOEU_Binomial <- subset(data_LOEU_Binomial, Outcome !=
"nonLOEU-CN")
data_LOEU_Binomial <- subset(data_LOEU_Binomial, Outcome !=
"nonLOEU-CI")
data_LOEU_Binomial$Outcome <- factor(data_LOEU_Binomial$Outcome,
levels = c("LOEU-CN", "LOEU-CI"), ordered = TRUE)
######### MICE Multiple Imputation
# Selecting the variables to be included in MICE dataset
Variables_overall <- c("Age", "Sex", "Edu_yrs", "Overweight", "BMI",
"Obesity", "Onset_year", "Active_Sz", "EpiDur", "BTCS", "Sleep.disorder",
"Mood.disorder", "HBP", "DM", "Lipidemia", "CHD", "Smoking", "Stroke",
"Trauma", "Surgery", "Cer_Infection", "Tumor", "Alcohol_Consume",
"Image_Pos", "Cerebral_Atrophy", "HS", "SVD", "Vascular_Mal", "Malacia",
"CurrMed", "EEG_Con", "SWA", "G.theta", "G.delta", "FS.left", "FS.right",
"FS.bilateral", "TIRDA", "TIRDA_total", "Ant_Tem_IED", "Oht_Tem_IED",
"Extra_Tem_IED")
formula <- as.formula(paste("Outcome ~", paste(Variables_overall, collapse =
" + ")))
data_LOEU_Binomial <- data_LOEU_Binomial[, c("Outcome",
Variables_overall)]
# Defining imputation methods
md.pattern(data_LOEU_Binomial)
vars_with_missing <-
names(data_LOEU_Binomial)[colSums(is.na(data_LOEU_Binomial)) > 0]
vars_without_missing <- setdiff(names(data_LOEU_Binomial),
vars_with_missing)
methods <- make.method(data_LOEU_Binomial)
methods["BMI"] <- "pmm"
```

```
methods["Obesity"] <- "logreg"
methods["Edu_yrs"] <- "pmm"
methods["Active_Sz"] <- "logreg"
methods["Image_Pos"] <- "logreg"
methods["Cerebral_Atrophy"] <- "logreg"
methods["HS"] <- "logreg"
methods["SVD"] <- "logreg"
methods["Vascular_Mal"] <- "logreg"
methods["Malacia"] <- "logreg"
pred_matrix <- make.predictorMatrix(data_LOEU_Binomial)
pred_matrix[, vars_without_missing] <- 0
# MICE
imp <- mice(data_LOEU_Binomial, m =10, method = methods, seed = 1234,
predictorMatrix = pred_matrix)
summary(imp)
########## LASSO regression for each of 10 datasets, manually record the
rate of non-zero coefficients
completed_data <- complete(imp, "i")    # "i" = c(1, 10)
x <- model.matrix(Outcome ~ . -1, data = completed_data)
y <- as.numeric(completed_data$Outcome) - 1
f = glmnet(x, y, family = "binomial", alpha = 1)
mod_cv <- cv.glmnet(x = x, y = y, family = "binomial", alpha = 1, type.measure
= "auc")
best_lambda <- mod_cv$lambda.min
lasso_model <- glmnet(x, y, family = "binomial", alpha = 1, lambda =
best_lambda)
coef(lasso_model)
######### Data selection based on the occurence rate of variables, pick ≥ 8
############
######### Re-run binomial logistic regression using the selected
combination of variables    ############

########## Calculate P-values and ORs
logit_model_LASSO <- with(imp, glm(Outcome ~ Age + EEG_Con + G.delta +
Ant_Tem_IED, family = binomial))
summary(logit_model_LASSO)
pooled_results <- pool(logit_model_LASSO)
summary(pooled_results)
summary_pooled_LASSO <- summary(pooled_results)
summary_pooled_LASSO$OR <- exp(summary_pooled_LASSO$estimate)

#  计算OR2

summary_pooled_LASSO$conf.low <-
exp(summary_pooled_LASSO$estimate - 1.96 *
```

```
summary_pooled_LASSO$std.error)   # 95% CI1下限
summary_pooled_LASSO$conf.high <-
exp(summary_pooled_LASSO$estimate + 1.96 *
summary_pooled_LASSO$std.error)   # 95% CI1上限
summary_pooled_LASSO$p.value <- sprintf("%.4f",
summary_pooled_LASSO$p.value)
summary_pooled_LASSO$OR <- sprintf("%.3f",
summary_pooled_LASSO$OR)
summary_pooled_LASSO$conf.low <- sprintf("%.3f",
summary_pooled_LASSO$conf.low)
summary_pooled_LASSO$conf.high <- sprintf("%.3f",
summary_pooled_LASSO$conf.high)
# Results
summary_pooled_LASSO[, c("term", "OR", "conf.low", "conf.high", "p.value")]

########## Calculate AUROC, sensitivity, specificity, npv, ppv, accuracy
# Re-run logistic regression
model_results1 <- list()
for (i in 1:10) {
  completed_data <- complete(imp, action = i)
  logit_model_LASSO <- glm(Outcome ~ Age + EEG_Con + G.delta +
Ant_Tem_IED,
                           data = completed_data, family = binomial)
  model_results[[i]] <- logit_model_LASSO
}
pooled_results <- pool(model_results)
# Calculating predicted probability
predicted_probs <- sapply(model_results, function(model) predict(model, type
= "response"))
average_predicted_probs <- rowMeans(predicted_probs)
# Performing ROC analysis
true_classes <- complete(imp, action = 1)$Outcome
roc_obj <- roc(true_classes, average_predicted_probs)
auc_value <- auc(roc_obj)
print(auc_value)
auc_ci <- ci(roc_obj, conf.level = 0.95)
# Calculating NPV, PPV, sensitivity, specificity, accuracy
best_threshold <- coords(roc_obj, "best", ret = "threshold", transpose =
FALSE)
best_threshold_value <- as.numeric(best_threshold[1])
average_predicted_probs <- as.vector(average_predicted_probs)
predicted_classes <- ifelse(average_predicted_probs >=
```

```
best_threshold_value, 1, 0)
print(length(predicted_classes))
print(length(true_classes))
confusion_matrix <- table(Predicted = predicted_classes, Actual =
true_classes)
TN <- confusion_matrix[1, 1]
FP <- confusion_matrix[1, 2]
FN <- confusion_matrix[2, 1]
TP <- confusion_matrix[2, 2]
n <- TN + FP + FN + TP
sensitivity <- TP / (TP + FN)
specificity <- TN / (TN + FP)
ppv <- TP / (TP + FP)
npv <- TN / (TN + FN)
accuracy <- (TP + TN) / n
sens_ci <- binom.test(TP, TP + FN)$conf.int
spec_ci <- binom.test(TN, TN + FP)$conf.int
ppv_ci   <- binom.test(TP, TP + FP)$conf.int
npv_ci   <- binom.test(TN, TN + FN)$conf.int
acc_ci   <- binom.test(TP + TN, n)$conf.int
cat(sprintf("Sensitivity: %.3f (95%% CI: %.3f - %.3f)\n", sensitivity, sens_ci[1],
sens_ci[2]))
cat(sprintf("Specificity: %.3f (95%% CI: %.3f - %.3f)\n", specificity, spec_ci[1],
spec_ci[2]))
cat(sprintf("PPV:         %.3f (95%% CI: %.3f - %.3f)\n", ppv, ppv_ci[1],
ppv_ci[2]))
cat(sprintf("NPV:         %.3f (95%% CI: %.3f - %.3f)\n", npv, npv_ci[1],
npv_ci[2]))
cat(sprintf("Accuracy:    %.3f (95%% CI: %.3f - %.3f)\n", accuracy, acc_ci[1],

acc_ci[2]))
```
